# A plant Lysin Motif Receptor-Like Kinase plays an ancestral function in mycorrhiza

**DOI:** 10.1073/pnas.2426063122

**Published:** 2025-06-11

**Authors:** Eve Teyssier, Sabine Grat, David Landry, Mathilde Ouradou, Mélanie K. Rich, Sébastien Fort, Jean Keller, Benoit Lefebvre, Pierre-Marc Delaux, Malick Mbengue

**Affiliations:** ^a^Laboratoire de Recherche en Sciences Végétales, Université de Toulouse, CNRS, Institut National Polytechnique Toulouse, Castanet-Tolosan 31320, France; ^b^Laboratoire des Interactions Plantes-Microbes-Environnement, Institut national de recherche pour l’agriculture, l’alimentation et l’environnement, CNRS, Université de Toulouse, Castanet-Tolosan 31326, France; ^c^Université Grenoble Alpes, CNRS, Centre de Recherche sur les Macromolécules Végétales, Grenoble 38000, France

**Keywords:** LysM-RLKs, arbuscular mycorrhiza, *Marchantia paleacea*

## Abstract

Arbuscular mycorrhiza (AM) with soilborne Glomeromycota fungi was pivotal in the conquest of land by plants almost half a billion years ago. In flowering plants, it is hypothesized that AM is initiated by the perception of AM fungi-derived chito- and lipochito-oligosaccharides (COs/LCOs) in the host via Lysin Motif Receptor-Like Kinases (LysM-RLKs). However, it remains uncertain whether plant perception of these molecules is a prerequisite for AM establishment and for its origin. Here, we made use of the reduced LysM-RLK complement present in the liverwort *Marchantia paleacea* to assess the conservation of the role played by this class of receptors during AM and in CO/LCO perception. Our reverse genetic approach demonstrates the critical function of a single LysM-RLK, MpaLYKa, in AM formation, thereby supporting an ancestral function for this receptor in symbiosis. Binding studies, cytosolic calcium variation recordings and genome-wide transcriptomics indicate that another LysM-RLK of *M. paleacea*, MpaLYR, is also required for triggering a response to COs and tested LCOs, despite being dispensable for AM formation. Collectively, our results demonstrate that the perception of symbionts by LysM-RLK is an ancestral feature in land plants, and suggest the existence of yet-uncharacterized AM fungi signals.

## Significance

Terrestrial plants associate with symbiotic fungi to acquire nutrients. In flowering plants this symbiosis is thought to depend on the perception of specific fungal-derived molecules by plant receptors called Lysin Motif-Receptor-Like Kinases (LysM-RLKs). Here, we show that the perception of these molecules in the nonvascular plant *Marchantia paleacea* also requires two LysM-RLKs, demonstrating the ancestral nature of this symbiotic dialogue. However, only one of the two receptors is crucial for symbiosis. We conclude that LysM-RLK-mediated perception of arbuscular mycorrhiza (AM) fungi is an ancestral requirement for AM symbiosis and suggest the existence of yet to be discovered symbiotic signal(s).

To ensure their mineral nutrition, most land plants form arbuscular mycorrhiza (AM) with soilborne Glomeromycota fungi (1). This ~450 My old symbiosis played a crucial role in driving the colonization of land by plants (2). In angiosperms, as dicots including legumes, AM is thought to be initiated via the perception by the host plant of AM fungi derived chito-(COs) and lipochito-oligosaccharides (LCOs) leading to the activation of a conserved signaling pathway referred to as the Common Symbiosis Signaling Pathway (CSSP) (3). Mutation of any CSSP gene, such as the receptor-like kinase *SYMRK* (4), leads to the inability to associate with AM fungi in species as diverse as legumes (4, 5), monocots (6, 7), or bryophytes (8, 9).

Genetics in legumes and monocots have demonstrated that members of the diversified Lysin Motif Receptor-Like Kinase (LysM-RLK) family, which encompasses up to 22 members in legumes (10), are important for the perception of these AM fungi derived molecules (11–13). Only one double mutant in the legumes *Medicago truncatula* and *Lotus japonicus* showed a phenotype phenocopying mutants of the CSSP with a total lack of colonization, demonstrating the importance of this family of receptors for AM in flowering plants (14, 15). However, none of the mutant combinations fully abolished the perception of both COs and LCOs, thus not allowing to determine whether these symbiotic signals are sufficient for AM establishment or whether other signal(s) are involved. The LysM-RLK family expansion that occurred in angiosperms likely complicates such analysis due to genetic redundancy.

Unlike angiosperms, such as legumes and monocots, which are vascular plants, bryophytes are nonvascular plants. This group includes species with a haploid-dominant life-cycle, such as the liverwort *Marchantia paleacea*. Because the bryophytes and vascular plant lineages diverged from each other soon after the colonization of lands by plants, any trait found to be conserved in both clades can be inferred as ancestral in plants (16, 17). Using reverse genetics in *M. paleacea* and comparison with vascular plants, it has been demonstrated that AM has been relying on lipids provided by the host plants for 450 My (18). Furthermore, mutations in any of the CSSP gene in *M. paleacea* lead to the total absence of colonization by AM fungi, as observed in angiosperms (8, 19). It can thus be hypothesized that at least part of the signaling processes involved in the formation of AM in *M. paleacea* and angiosperms are similar. In contrast to angiosperms, the liverwort *M. paleacea* contains only four LysM-RLKs (see below), limiting the expected genetic redundancy observed in angiosperms. *M. paleacea* therefore represents a unique model to study the role of CO- and LCOs-signaling and determine whether other signals are involved in AM establishment.

In this study, we demonstrate the essential role for AM in *M. paleacea* of a single LysM-RLK, pro-ortholog to the highly diversified CERK1 clade in most angiosperms. Moreover, we demonstrate that the ability of *M. paleacea* to respond to COs and LCOs is fully abolished in that mutant. Yet, we present genetic evidence that signaling triggered by COs and LCOs might not be essential for AM in *M. paleacea*, suggesting the existence of additional and uncharacterized AM fungi signals.

## Results

### *M. paleacea* Has a Limited Number of LysM-RLKs.

**Fig. 1.**
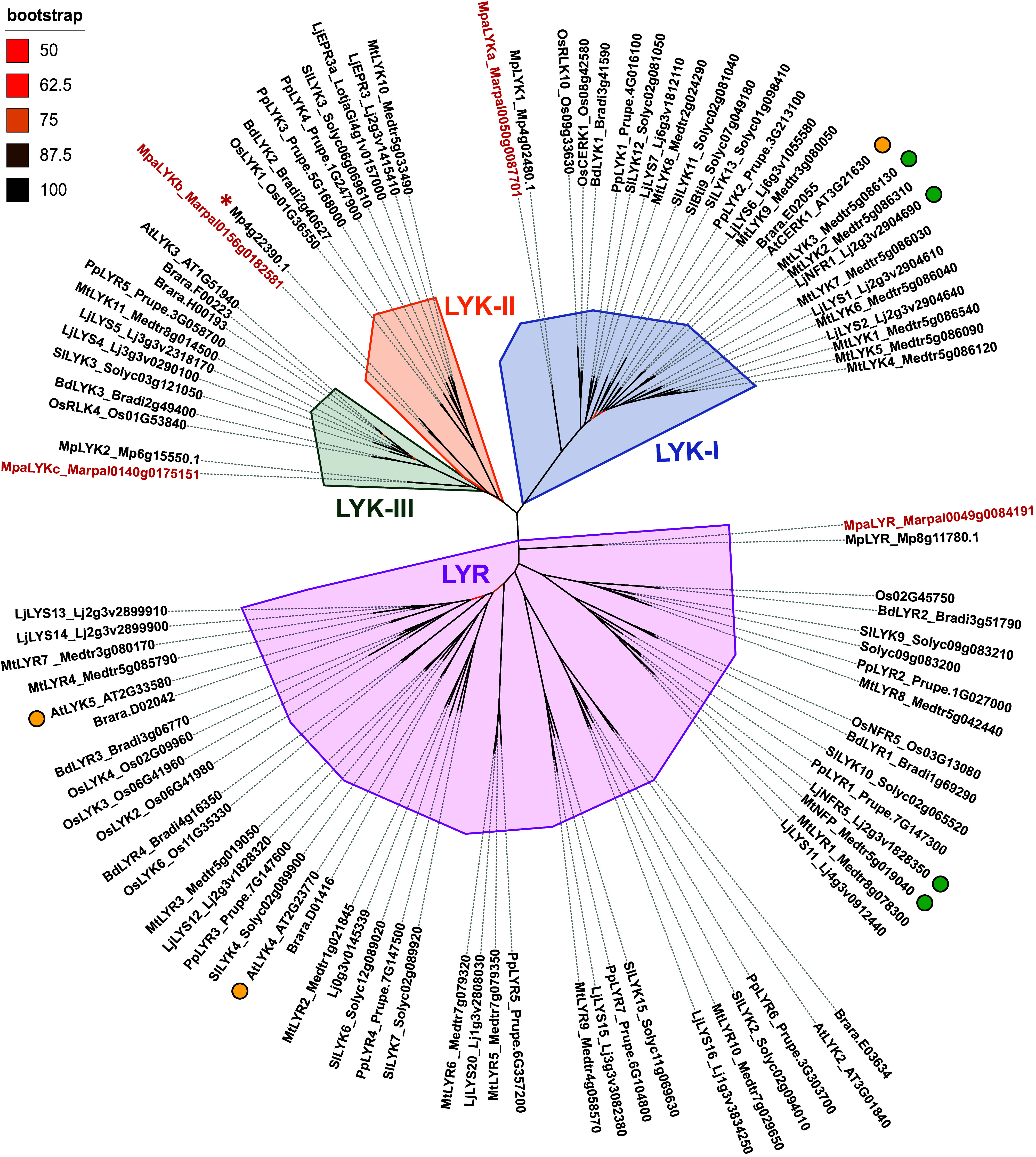
*M. paleacea* possesses four LysM Receptor-Like Kinases. Phylogenetic analysis was performed using a set of 102 LysM-RLKs from land plant species. Full-length protein sequences from *Arabidopsis thaliana* (At_AT)*, Brassica rapa* (Brara)*, Solanum lycopersicum* (Sl_Solyc), *Oriza sativa* (Os), *Prunus persica* (Pp_Prupe), *Brachypodium distachyon* (Bd_Bradi), *Lotus japonicus* (Lj_Lj/Lotja), *Medicago truncatula* (Mt_Medtr), *M. polymorpha* (Mp), and *M. paleacea* (Mpa_Marpal) were aligned with MUSCLE prior unrooted Maximum Likelihood phylogenetic tree construction using 1000 bootstraps resampling value. Branch colors indicate bootstrap values. Four phylogenetic subclades are highlighted: LYK-I (blue), LYK-II (orange), LYK-III (green), LYR (pink). *M. paleacea* proteins are labeled in red. The red star sign indicates the truncated homolog of MpaLYKb in *M. polymorpha*. Notable receptors for long-chain CO perception in *Arabidopsis* or Nod-LCOs perception in legumes are indicated with yellow or green dots, respectively.

Phylogenetic analyses indicate that LysM-RLK originated in streptophyte green algae and diversified following the colonization of land by plants (20, 21). Extant liverworts harbor four clades, including three LYKs (LysM domain-containing RLK) with a predicted active kinase and one LYR (LYK-related) with a deleted activation loop in the kinase domain (22). All LysM-RLKs of *M. paleacea* contain a predicted N-terminal signal peptide, a LysM-containing extracellular domain, a transmembrane region, and an intracellular kinase domain. Based on the established nomenclature for LysM-RLKs (10), *M. paleacea* LYKa belongs to the LYK-I subclade that contains the immunity-related chitin receptor CERK1 from *Arabidopsis thaliana* (23) and the symbiotic Nod factor receptor NFR1 from *L. japonicus* (24) (Fig. 1). As a unique member, MpaLYKa therefore represents the pro-ortholog in *M. paleacea* of all members of the LYK-I subclade in angiosperm. Similarly, MpaLYKb and MpaLYKc are pro-orthologous to the LYK-II and LYK-III subclades, respectively, while the single MpaLYR is pro-orthologous to the entire LYR-I to LYR-IV subclades (Fig. 1). *Marchantia polymorpha*, that has lost the ability to form AM (25), contains only two *LYKs* (from the LYK-I and LYK-III subclades) and one *LYR* (22). The presence of a third LYK gene in *M. polymorpha*, which encodes a truncated LYK-II receptor devoid of its extracellular region, accounts for the observed discrepancy in LysM-RLK numbers between Marchantia species (Fig. 1).

### Mutation of a Single *LYK* Gene in *M. paleacea* Leads to Symbiosis Impairment.

**Fig. 2.**
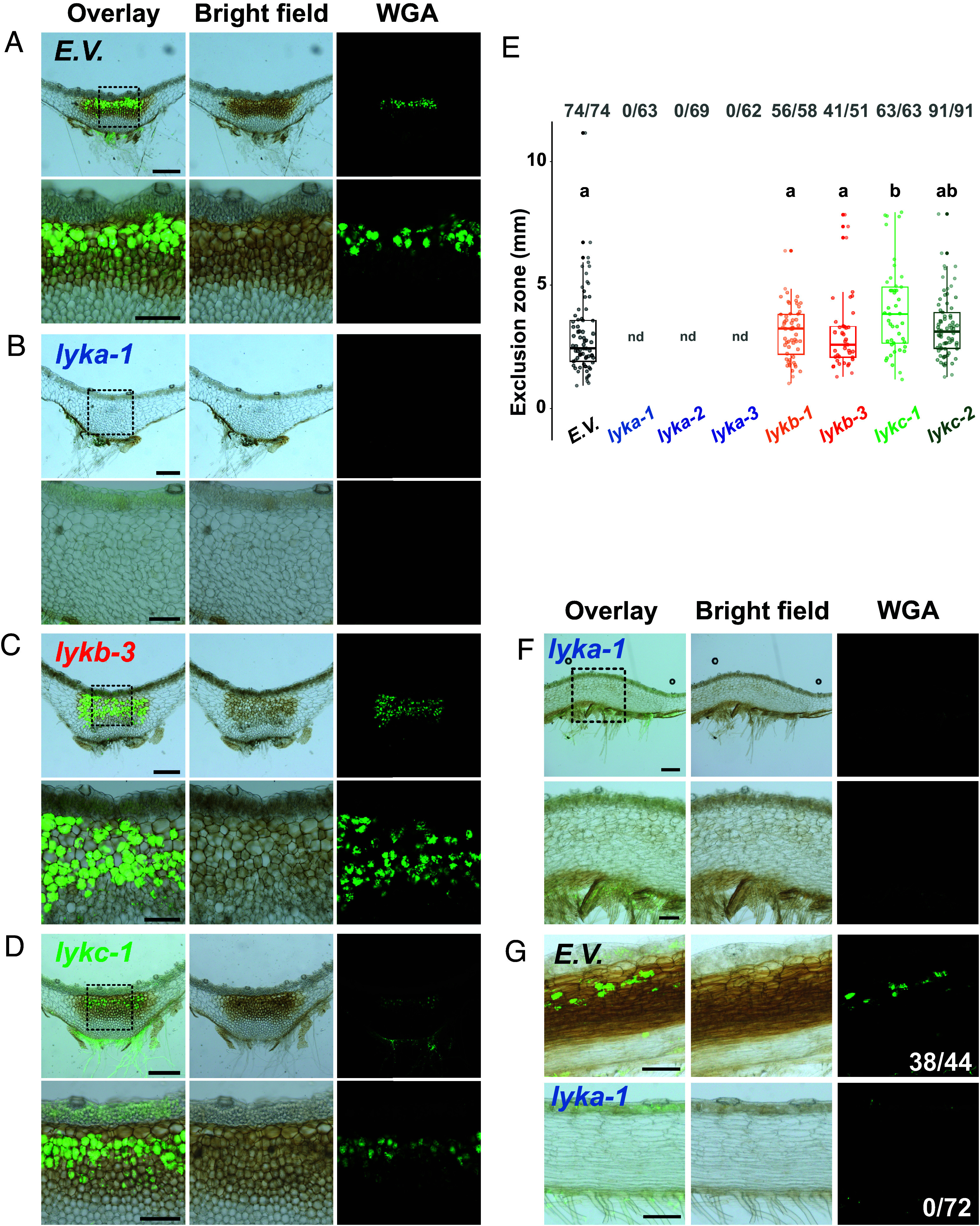
*MpaLYKa* is essential for arbuscular mycorrhiza in *M. paleacea.* (*A*–*D*) Representative images of transversal sections of *M. paleacea* control (E.V.) or *lyk* mutants, as indicated, 6 wk after inoculation with *R. irregularis*. *Left* panels are overlays of bright field (*Middle*) and fluorescent (*Right*) images. Wheat germ agglutinin (WGA) coupled to Alexa Fluor 488 was used to detect fungal structures. Dashed line delimited *Insets* are enlarged underneath the original images. Scale bars are 500 µm and 200 µm for *Insets*. (*E*) Quantitative analysis of the exclusion zone length on mycorrhizal plants for control (E.V.) and two independent loss-of-function mutants for each *LYK*. Fractions in bold gray represent mycorrhizal thalli over total thalli assessed for each genotype. Different letters indicate differences to control inferred by ANOVA followed by Tukey’s HSD post hoc test (*P*-value < 0.05). Control (E.V.) values are shared with Fig. 3 and statistical analysis was performed on the whole dataset. “nd” stands for not determined. (*F*) Longitudinal sections of the *lyka-1* mutant 10 wk after inoculation with *R. irregularis*. (*G*) Longitudinal sections of *M. paleacea* control (E.V.) or a *lyka-1* mutant 6 wk after cocultivation in close proximity with wild-type mycorrhizal *M. paleacea* nurse plants. Numbers of mycorrhizal thalli over total thalli tested are indicated. (Scale bar, 200 µm.)

To determine the contribution of individual LysM-RLKs from the *M. paleacea* LYK clade to AM, we generated loss-of-function mutants for *MpaLYKa*, *MpaLYKb* or *MpaLYKc*. For that, CRISPR/Cas9 was used in combination with single guide-RNAs (sgRNA) designed to target the 5’ region of the genes, hereby breaking the link between the signal peptide and the remaining part of these type-I membrane proteins (*SI Appendix*, Fig. S1). Following plant transformation and subsequent progeny selection, we recovered independent mutant lines for each *LYK* gene. Sanger sequencing of induced mutations are summarized in *SI Appendix*, Table S1. For *MpaLYKa* and *MpaLYKb*, only one of the two sgRNAs proved effective in inducing frameshift-causing INDELs whereas the two different sgRNAs designed against *MpaLYKc* were effective in inducing null mutations. For each LYK, we kept a minimum of two null mutant lines resulting from independent T-DNA transformation events and assessed their ability to form AM in comparison to an empty vector-containing control line. Six weeks after inoculation with spores of the AM fungus *Rhizophagus irregularis*, we evaluated the colonization status of *M. paleacea* by histological observations of transversal (Fig. 2 *A*–*D*) or longitudinal (*SI Appendix*, Fig. S2 *A*–*D*) sections of the thallus. A strong pigmentation along the midrib of the thallus is a marker of a positive mycorrhizal colonization in *M. paleacea* (26). This pigmentation was visible in control, *lykb,* and *lykc* mutants but absent in *lyka*, suggesting successful symbiosis establishment in *lykb* and *lykc* and a lack of fungal colonization in *lyka* (Fig. 2 *A*–*D* and *SI Appendix*, Fig. S2 *A*–*D*, *Middle*). Wheat-Germ-Agglutinin (WGA)-Alexa Fluor staining of fungal cell wall confirmed the presence of arbuscules in the thalli of control, *lykb,* and *lykc* plants. In contrast, no intercellular hyphae nor arbuscules were observed in *lyka* (Fig. 2 *A*–*D* and *SI Appendix*, Fig. S2 *A*–*D*, *Left* and *Right*). Over two large scale experiments, quantitative analysis of the exclusion zone length (2) as well as the percentages of mycorrhizal plants per genotype did not reveal differences between *lykb* and *lykc* alleles compared to the control (Fig. 2*E* and *SI Appendix*, Fig. S2*E*). In contrast, these experiments confirmed AM-defectiveness in three independent *lyka* mutants, representing approximately 300 measurements in total for this class of mutant.

To verify that the absence of fungal colonization in *lyka* was not due to a delay in AM establishment, we harvested *lyka* 10 wk after inoculation with *R. irregularis* spores. Again, we were unable to observe accumulation of the AM-related pigment or positive WGA-Alexa Fluor signal in the thalli of inoculated *lyka* plants (Fig. 2*F*). Next, we tested for complementation of *lyka* when cocultivated in proximity to wild-type *M. paleacea* that had been hosting AM fungi for several weeks. This nurse-plant method has been successfully used to distinguish between defaults in penetration and in survival of AM fungi in mutants. Six weeks after the start of the coculture with nurse plants, the empty-vector control plants showed an 86% success rate (38/44) in fungal colonization (Fig. 2*G*). Conversely, none of the *lyka* plants grown under these conditions were colonized (Fig. 2*G*), indicating that the *lyka* phenotype is not due to a secondary effect on the fungal symbiont but is rather the direct consequence of a nonfunctional MpaLYKa-dependent signaling pathway in the host. We therefore conclude that, within the *LYK* clade of *M. paleacea*, *MpaLYKa* is critical for AM establishment whereas *MpaLYKb* and *MpaLYKc* are dispensable.

### The Single LYR Gene from *M. paleacea* Is Not Essential for Arbuscular Mycorrhiza.

**Fig. 3.**
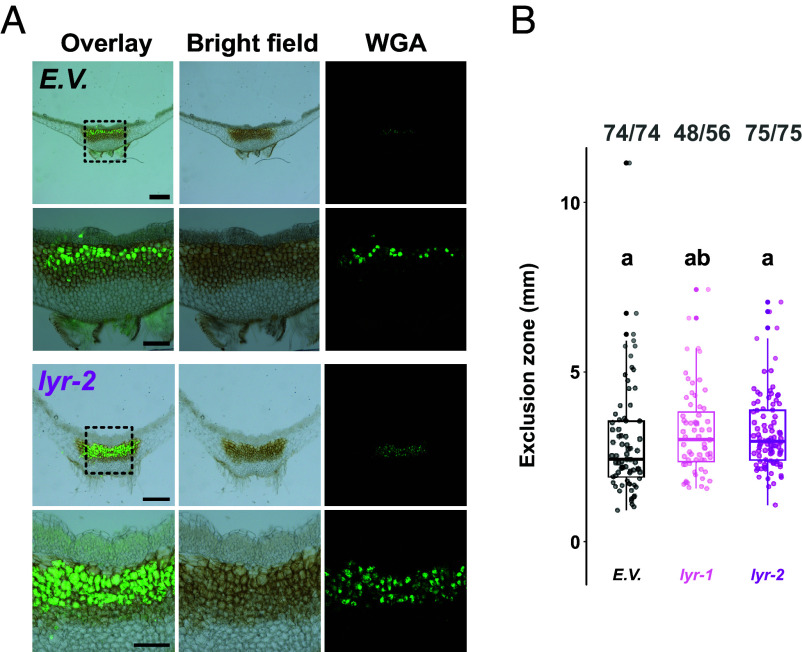
*MpaLYR* is dispensable for arbuscular mycorrhiza in *M. paleacea*. (*A*) Representative images of transversal sections of *M. paleacea* control (E.V.) or the *lyr-2* mutant 6 wk after inoculation with *R. irregularis*. *Left* panels are overlays of bright field (*Middle*) and fluorescent (*Right*) images. Wheat germ agglutinin (WGA) coupled to Alexa Fluor 488 was used to detect fungal structures. Dashed line delimited *Insets* are enlarged underneath the original images. Scale bars are 500 µm and 200 µm for *Insets*. (*B*) Quantitative analysis of the exclusion zone length on mycorrhizal plants for control (E.V.) and two *lyr* mutants. For each genotype, fractions in bold gray represent mycorrhizal thalli over total thalli assessed. Different letters indicate differences to control inferred by ANOVA followed by Tukey’s HSD post hoc test (*P*-value < 0.05). Control values are shared with Fig. 2 and statistical analysis was performed on the whole dataset.

Contrary to AM-host angiosperms, which carry between 4 and 11 LYR paralogs (10), the genome of *M. paleacea* contains a single *LYR* gene (25) (Fig. 1). To assess its contribution to symbiosis, we generated *lyr* loss-of-function alleles using CRISPR/Cas9 and two independent sgRNAs, as previously described (*SI Appendix*, Fig. S1 and Table S1), and tested their ability to form AM. As performed for the *lyk* mutants, we evaluated the colonization status of the *lyr* mutants by histological observations of transversal (Fig. 3*A*) or longitudinal (*SI Appendix*, Fig. S3*A*) sections of the thallus. We observed no differences in thallus pigmentation between empty-vector control and *lyr*, suggesting similar colonization levels in both genotypes. WGA-staining confirmed the presence of arbuscules in both control and *lyr* lines (Fig. 3*A* and *SI Appendix*, Fig. S3*A*). Similarly to what we observed for the *lykb* and *lykc* mutants, quantitative analysis of the exclusion zone length (2) as well as the percentages of mycorrhizal plants per genotype did not point at reproducible differences between *lyr* alleles and control (Fig. 3*B* and *SI Appendix*, Fig. S3*B*). Over two large scale experiments, control plants displayed a total of 98% mycorrhizal success rate and *lyr* alleles followed closely with 92% and 96% mycorrhizal success rate. The mean lengths of the exclusion zone were similar between genotypes, although a slight difference of approx. 1 mm was measured across replicates (Fig. 3*B* and *SI Appendix*, Fig. S3*B*). The absence of AM phenotype in mutants of the single *LYR* gene demonstrates the nonessential role for this clade of LysM-RLK for AM establishment in *M. paleacea*.

### MpaLYR Is the CO/LCO Receptor of *M. paleacea*.

**Fig. 4.**
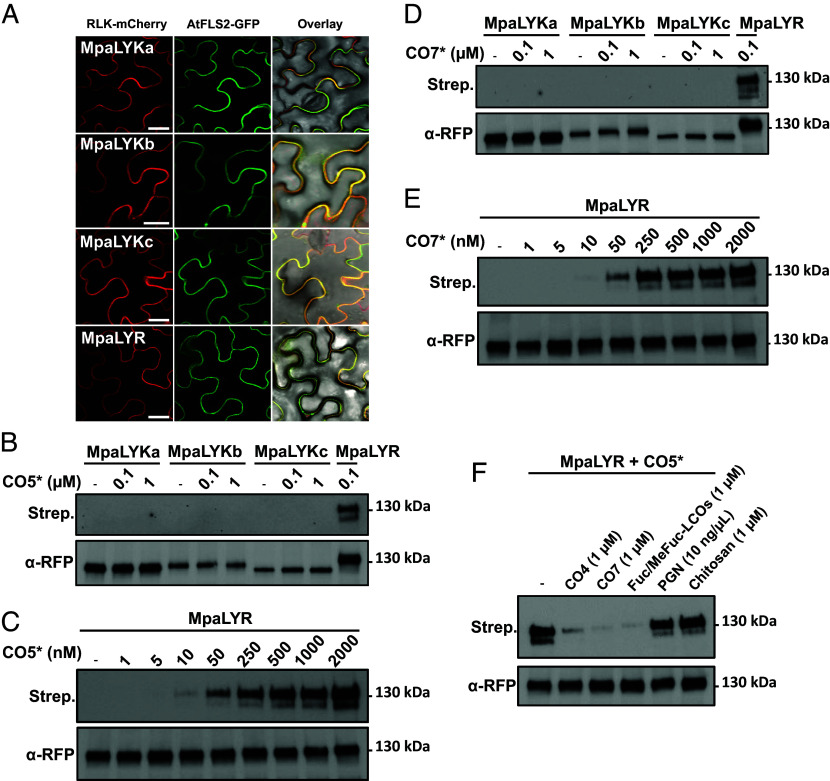
MpaLYR has high affinity for chito- and lipochito-oligosaccharides in *M. paleacea.* (*A*) Confocal micrographs of *N. benthamiana* epidermal cells transiently coexpressing indicated *M. paleacea* LysM-RLK-mCherry fusions (*Left*) and the plasma membrane marker AtFLS2-GFP (*Middle*). Overlays including bright field images are shown (*Left*). (Scale bar, 25 µm.) (*B*–*F*) Microsomal fractions from *N. benthamiana* leaves expressing *M. paleacea* LysM-RLK-mCherry fusions, as indicated, were utilized in binding assays with indicated concentrations of crosslinkable biotinylated chitopentaose (CO5*) or chitoheptaose (CO7*). Ligand bound receptors and total immunopurified receptors were detected using Streptavidin (*Top*) or anti-RFP antibodies (*Bottom*), respectively. (*F*) CO5*-binding competition assays on MpaLYR using 10-fold higher concentrations of CO4, CO7, Fuc/MeFuc-LCOs, PGN fragments, or chitosan fragments relative to CO5*.

In order to gain insights into the functional roles of *M. paleacea* LysM-RLKs in the perception of COs and LCOs, we conducted binding assays using tagged receptors transiently expressed in *Nicotiana benthamiana* cells. First, the subcellular localization of the four receptor proteins tagged in their C-terminus with mCherry was verified by confocal microscopy when coexpressed with the well-known plasma-membrane resident *Arabidopsis* Flagellin Sensing 2 (FLS2) receptor which was fused with GFP (27, 28) (Fig. 4*A*). For each receptor, the mCherry signal was visible on the epidermal cell periphery and perfectly superimposed with the GFP signal, demonstrating the correct trafficking for all four *M. paleacea* LysM-RLK fusion constructs to the plasma membrane in *N. benthamiana* (Fig. 4*A*). Next, microsomal preparations from leaf tissue expressing single LysM-RLK were mixed with increasing concentrations of crosslinkable biotinylated chito-pentaose or chito-heptaose (hereafter CO5* and CO7*, respectively) before detection of the biotin moieties and the mCherry tag by Western blot (Fig. 4 *B* and *D*). While none of the LYK receptors exhibited binding to CO5* or CO7* at concentrations up to 1 μM, a clear signal was observed for MpaLYR when challenged with only 100 nM of CO5* or CO7* (Fig. 4 *B* and *D*). The overexposure of the streptavidin blots did not reveal any weak signals, thereby suggesting that neither MpaLYKa, MpaLYKb nor MpaLYKc are able to bind CO5* or CO7* in these conditions (*SI Appendix*, Fig. S4). To determine the affinity of the MpaLYR receptor with regard to COs, experiments were conducted in which various concentrations of CO5* and CO7*, ranging from 1 nM to 2 μM, were tested (Fig. 4 *C* and *E*). The presence of ligand-bound LYR proteins was observed at concentrations as low as 10 nM of CO5* or 50 nM of CO7* (Fig. 4 *C* and *E*). Saturation of the signal between 250 and 500 nM indicates that the LYR receptor exhibits a high affinity for both short and long CO molecules. To confirm these findings and test whether MpaLYR could also bind LCOs, a competition assay was performed wherein MpaLYR-containing microsomes were simultaneously incubated with 100 nM CO5* and 10-fold higher concentration (1 µM) of unlabeled chito-tetraose (CO4), CO7, or the major forms of LCOs produced by *R. irregularis*, LCO-V (C18:1, Fuc/MeFuc), hereafter Fuc/MeFuc-LCOs (29) (Fig. 4*F*). Additionally, 1 µM of deacetylated chitin (chitosan) fragments or 10 ng µL^−1^ peptidoglycan fragments derived from *Bacillus subtilis* were used as negative controls. The application of unlabeled CO4, CO7, or Fuc/MeFuc-LCOs resulted in a strong reduction in the binding of CO5* to MpaLYR when compared to the control condition in the absence of competitors. In contrast, the application of bacterial peptidoglycan or chitosan fragments had no discernible effect on the binding of CO5* to the MpaLYR receptor (Fig. 4*F*). Collectively, these results support the conclusion that MpaLYR serves as a CO and LCO receptor.

### COs and LCOs Early Signaling Is Impaired in *lyka* and *lyr* Mutants of *M. paleacea*.

The function of MpaLYR, but not MpaLYKa, as a receptor for COs and LCOs was in apparent contradiction with the AM phenotypes of the corresponding mutants, with *MpaLYKa* but not *MpaLYR*, being critical for AM establishment. Therefore, we sought to correlate AM establishment and perception of AM fungus-derived signals by determining the signaling abilities of the different *M. paleacea* receptor mutants in response to COs and LCOs. Ligand perception events by receptor-like kinases induce within seconds calcium influx into the cytosol in both immune and symbiotic contexts (30). To use this physiological output, we generated a *M. paleacea* line expressing the cytosolic apoaequorin luminescent marker (31) under the control of the constitutive *MpoEF1α* promoter (32). Once established and tested for suitable apoaequorin expression, this line served as a homogeneous background to re-create loss-of-function mutants using CRISPR/Cas9, as well as a control line expressing the Cas9 endonuclease alone (AEQ-cas9). Plants were then treated with CO7, CO4, or different LCOs, including Fuc/MeFuc-LCOs, LCO-IV(C18:1) (hereafter NS-LCOs for nonsulfated-LCOs) and LCO-IV(C18:1,S) (hereafter S-LCOs for sulfated-LCOs) (29, 33).

**Fig. 5.**
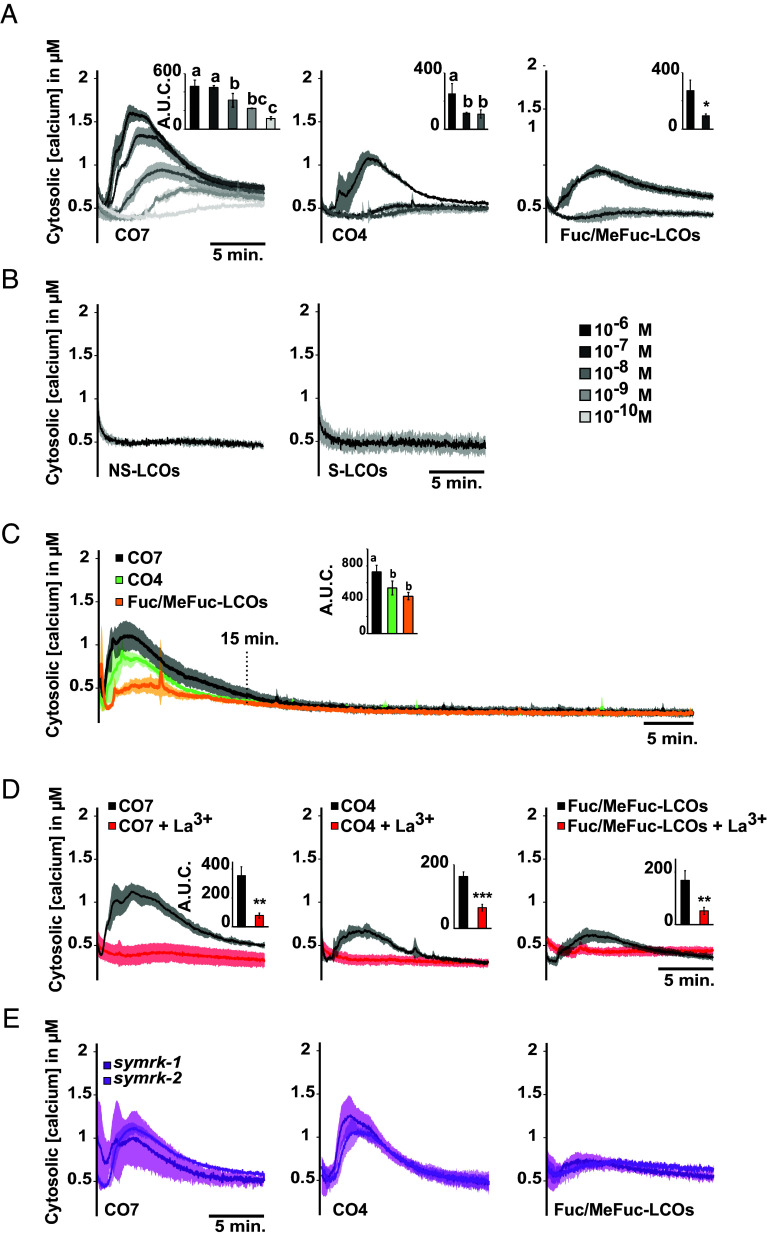
Chito- and lipochito-oligosaccharides induce cytosolic calcium influx in *M. paleacea* independently of the common symbiosis pathway. Cytosolic calcium concentration variations in *M. paleacea* control plants (AEQ-cas9) in response to decreasing concentrations of CO7, CO4, and Fuc/MeFuc-LCOs, as indicated, over a 15 min time course. (*B*) Cytosolic calcium concentration variations in *M. paleacea* control plants (AEQ-cas9) in response to 1 µM nonsulfated LCO (NS-LCO) or sulfated LCO (S-LCO), as indicated. (*C*) Cytosolic calcium concentration variations in *M. paleacea* control plants (AEQ-cas9) in response to 1 µM CO7, CO4, or Fuc/MeFuc-LCOs over a 1-h time course. (*D*) Effect of a 5 min pretreatment with 1.5 mM La^3+^, on CO7-, CO4-, or Fuc/MeFuc-LCO-induced calcium concentration variations. (*E*) Cytosolic calcium concentration variations in two independent *M. paleacea symrk* mutant lines in response to 1 µM CO7, CO4, or Fuc/MeFuc-LCOs. (*A*–*D*) Bar graph *Insets* represent the A.U.C. corrected for the baseline and different letters indicate significant differences to control conditions inferred by ANOVA followed by Tukey’s HSD post hoc test (*P*-value < 0.05) for (*A* and *C*) or Student *t* test (*P*-value < 0.01**; < 0.001***) for (*D*). (*A–E*) Each trace represents the mean (line) ± SD (shading) from at least three replicates.

To determine an effective working concentration for all molecules, we tested a range of concentrations starting at 10^−6^ M (1 µM) followed by 10-fold series dilutions (Fig. 5*A*). At the concentration of 1 µM, CO7, CO4, and Fuc/MeFuc-LCOs induced an increase in cytosolic calcium concentration in the *M. paleacea* control line that peaked at 5 min after application, before reaching back to the baseline at 15 min (Fig. 5*A*). In contrast, NS-LCOs and S-LCOs failed to elicit a calcium response at this concentration (Fig. 5*B*), suggesting variability in the potency of different LCO species. Importantly, water and dimethyl sulfoxide (DMSO) used to prepare stock solutions for CO4 and CO7 or LCOs, respectively, did not trigger any calcium variation in *M. paleacea* (*SI Appendix*, Fig. S5). Of the three active molecules, CO7 elicited the most pronounced response that gradually decreased in intensity and showed a slight delay when using diluted solutions. A clear response was still visible at 1 nM of CO7, although not statistically different from the lowest tested and poorly active concentration of 0.1 nM (Fig. 5 *A*, *Insets*). In contrast, CO4 and Fuc/MeFuc-LCOs already failed to elicit a clear response at 0.1 µM, as evidenced by the shapes of the corresponding response curves (Fig. 5*A*). In the following experiments, we have therefore chosen the highest concentration of 1 µM for all molecules. To verify whether cytosolic calcium variations occur at later time points, plant responses to CO7, CO4, and Fuc/MeFuc-LCOs were monitored over a 1-h time course. No additional calcium variation was apparent at later time points, suggesting that a 15 min time frame was sufficient to capture the entire cytosolic calcium response of *M. paleacea* to these molecules (Fig. 5*C*). A 5 min pretreatment with 1.5 mM lanthanum chloride (La^3+^), a nonspecific calcium channel blocker (34), completely abolished the cytosolic calcium variations triggered by CO7, CO4, or Fuc/MeFuc-LCOs (Fig. 5*D*). This supports the view that these cytosolic calcium variations are bona fide influxes of calcium originating from the apoplast.

To determine whether this calcium influx triggered by CO7, CO4, and Fuc/MeFuc-LCOs seat upstream or downstream of the CSSP, we analyzed the response of two independent *symrk* mutants generated in the aequorin-expressing *M. paleacea* background (*SI Appendix*, Fig. S1 and Table S1). The duration and amplitude of the calcium influxes measured in AEQ-*symrk-1* and AEQ-*symrk-2* after 1 µM CO7, CO4, or Fuc/MeFuc-LCOs treatments were similar to the control (Fig. 5*E*). This result demonstrates that this response is upstream SYMRK and distinct from calcium variations known as calcium spiking, which are strictly dependent on CSSP activation. Taken together, our results show that CO7, CO4, and Fuc/MeFuc-LCOs induce an early calcium influx in *M. paleacea* and that this physiological response depends on a perception module that is independent of SYMRK.

**Fig. 6.**
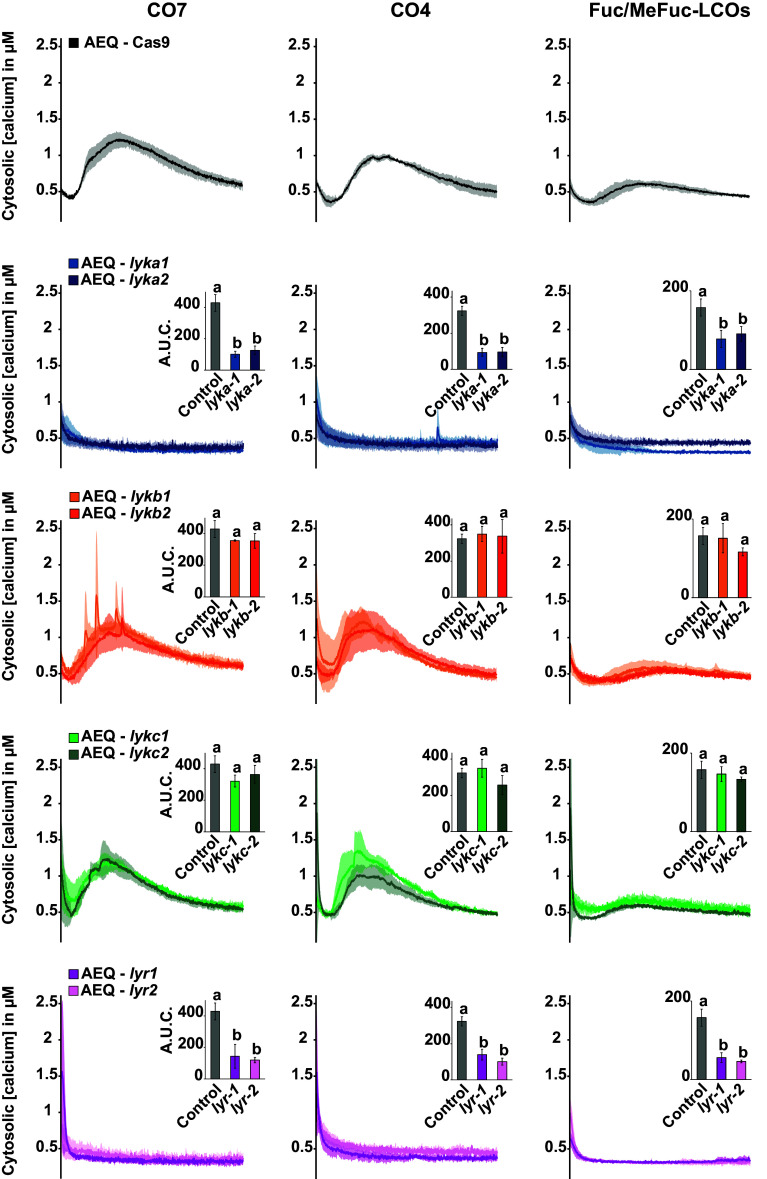
Chito- and lipochito-oligosaccharides-induced calcium influx response in *M. paleacea* depend on *MpaLYKa* and *MpaLYR*. Cytosolic calcium concentration variations in *M. paleacea* control (AEQ-cas9) or two independent mutant lines for each *LysM-RLK* of *M. paleacea*, as indicated. Plants were treated with 1 µM CO7 (*Left*), 1 µM CO4 (*Middle*), or 1 µM Fuc/MeFuc-LCOs (*Right*). Each trace represents the mean (line) ± SD (shading) from at least three replicates over a time course of 15 min. Bar graph *Insets* represent the A.U.C. corrected for the baseline and different letters indicate significant differences versus control inferred by ANOVA and Tukey’s HSD post hoc test (*P*-value < 0.05).

Having established some characteristics of this CO7-, CO4-, and Fuc/MeFuc-LCOs-induced calcium influx in *M. paleacea*, we further investigated the function of each LysM-RLK in generating this response. For that, we recovered two independent CRISPR/cas9-edited *M. paleacea* lines for each receptor encoding gene in the aequorin-expressing background (*SI Appendix*, Table S1). Next, we treated these plants with 1 µM CO7, CO4, or Fuc/MeFuc-LCOs and monitored their calcium influx response in comparison to the AEQ-cas9 control line (Fig. 6). The duration and amplitude of the calcium influxes obtained in AEQ-*lykb* and AEQ-*lykc* lines were comparable to the control for all three elicitors, whereas AEQ-*lyka* and AEQ-*lyr* lines failed to respond to all treatments (Fig. 6). Quantitative analysis of the area under the curves (A.U.C.) and ANOVA confirmed the similarity between control, AEQ-*lykb*, and AEQ-*lykc* and the differences between control and AEQ-*lyka* or AEQ-*lyr* in response to all elicitors (Fig. 6, bar graph *Insets*). To rule out the possibility that *lyka* and *lyr* mutations have a pleotropic effect on the plants ability to mount a calcium response, we treated all genotypes with hydrogen peroxide (H_2_O_2_) at a final concentration of 1 mM (*SI Appendix*, Fig. S6). All aequorin-expressing LysM-RLK mutants responded with an immediate and robust calcium influx and quantitative analysis of the A.U.C. and ANOVA confirmed that there was no difference between control, AEQ-*lyka,* and AEQ-*lyr* mutants in response to H_2_O_2_. In addition, the AM fungi colonization status of AEQ-*lyka* and AEQ-*lyr*, the two nonresponsive mutants to COs and Fuc/MeFuc-LCOs, was evaluated after *R. irregularis* inoculation and histological observations confirmed that AEQ-*lyka* was AM-defective (*SI Appendix*, Fig. S7). Conversely, as previously described (Figs. 2 and 3 and *SI Appendix*, Figs. S2 and S3), arbuscules were clearly visible in AEQ-cas9 and AEQ-*lyr* plants with no differences in the size of the exclusion zone (*SI Appendix*, Fig. S7).

Collectively, these results demonstrate that MpaLYKa and MpaLYR are both required for the perception of chitin-based molecules and their early signaling in *M. paleacea*.

### CO7-Induced Transcriptomic Changes Are Similarly Affected in *lyka* and *lyr* Mutants.

**Fig. 7.**
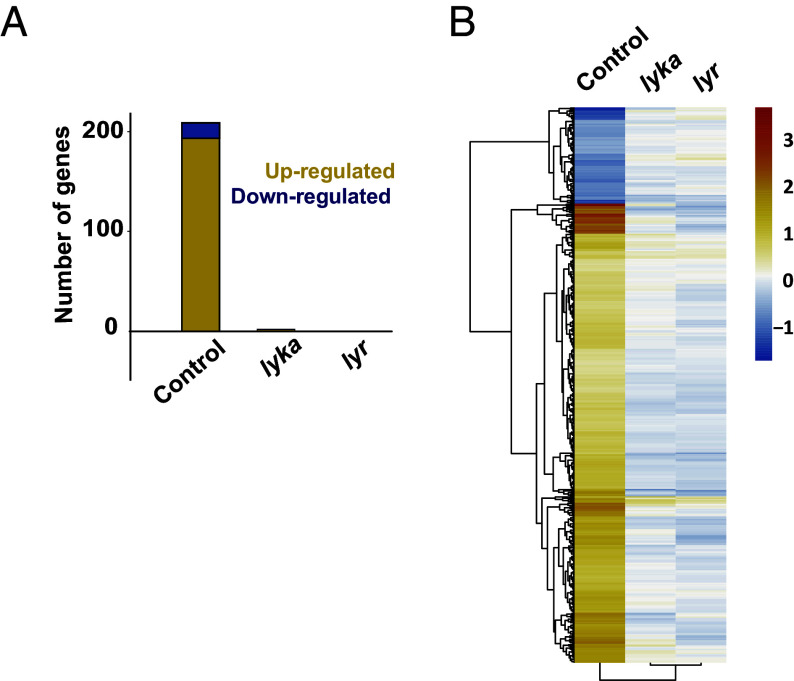
*MpaLYKa* and *MpaLYR* are essential for gene induction in response to CO7. (*A*) Number of significantly upregulated or downregulated genes (yellow or blue, respectively) in CO7-treated plants for 1-h (control, *lyka,* and *lyr*) relative to nontreated plants. DEGs are statistically significant (*P*-value adjusted < 0.05 and |log2FC| ≥ 1). (*B*) Heatmap of the log2 fold values in *lyka* and *lyr* mutants compared with the control in plants treated with 1 µM CO7. Displayed genes are DEGs for the control (*P*-value adjusted < 0.05).

The perception of nonself and the activation of corresponding signaling pathways in plants often culminate in adaptive transcriptomics reprogramming. Having established that early signaling in response to fungal molecules was blocked in both the *lyka* and *lyr* mutants, we then tested whether a later response such as gene induction was also affected in these mutants. First, we performed genome-wide transcriptomics of *M. paleacea* control plants after a 1-h treatment with 1 µM of CO7 or CO4. When compared to treatment with water only, CO7 and CO4 induced a statistically significant differential expression for 444 and 108 genes (*P*-adjusted value ≤ 0.05), respectively (Dataset S1). Of the differentially expressed genes (DEGs) identified in response to CO7, 195 were up-regulated with a log2 fold induction (log2FC) greater than 1, while 16 were down-regulated with a log2FC less than -1 (Fig. 7*A*). In response to CO4, the application of identical log2FC cut-off values identified 45 genes that were up-regulated and two genes that were down-regulated (Dataset S1). Interestingly, 87% (94/108) of DEGs identified in response to CO4 are shared with CO7 (*SI Appendix*, Fig. S8*A*) and a direct comparison of the log2FC values in response to both treatments for the 108 CO4-responsive genes resulted in a Pearson’s correlation coefficient of 0.91 (*SI Appendix*, Fig. S8*B*). The overlap and strong correlation between DEGs groups suggest that the transcriptomic response to CO4 represents a subset of the response to CO7 in *M. paleacea*. Furthermore, CO7 appears to be a more potent elicitor of gene reprogramming in this species, as evidenced by the fourfold higher number of identified DEGs in response to this molecule. With the exception of *MpalRAM2c* upregulated in response to CO7 treatment, we did not detect significant changes in the regulation of genes associated with AM or response to symbiotic fungus.

In order to gain insight into the most represented molecular functions, an InterPro domain enrichment analysis was performed on the up- and down-regulated genes in response to COs (*SI Appendix*, Fig. S9 and Dataset S2). A total of 18 and 15 InterPro terms were found to be significantly enriched among the CO7- and CO4-up-regulated genes, respectively (*SI Appendix*, Fig. S10 and Dataset S2). Among those, seven terms were present in response to both treatments and related to leucine-rich repeats (IPR032675, IPR001611), protein kinases (IPR000719, IPR008271, IPR011009, IPR017441), and secretory peroxidase (IPR033905). Interestingly, the plant immunity-associated term “WRKY domain superfamily” (IPR036576) was found in response to both treatments, albeit only significantly enriched in response to CO7 (Dataset S2). Taken together, these results suggest that COs perception in *M. paleacea* induces genes involved in cell-surface signaling, the oxidative response, and transcription. Conversely, the set of 16 down-regulated genes following CO7 treatment was significantly enriched in genes with functions related to photosynthesis and carbon fixation (*SI Appendix*, Fig. S9*B*), suggesting a potential growth inhibition trade-off after CO7 perception. The two down-regulated genes after CO4 treatment were not sufficient to perform a similar enrichment analysis.

In view of the overlap between the CO7 and CO4 transcriptomic responses observed in the control plants, we assessed the response of the *lyka* and *lyr* mutants to CO7 only. In the *lyka* mutant, CO7 treatment resulted in a statistically significant differential expression for 47 genes, with only two genes being up-regulated and two genes being down-regulated when the previously described log2FC cut-off values were applied (Fig. 7*A*). Moreover, none of these four up- or down-regulated genes were present in the list of DEGs identified in the control plants (Dataset S1), suggesting that they may represent noise rather than a true signal. In the *lyr* mutant, CO7 did not elicit any response in comparison to the water treatment, as none of the two DEGs identified in this mutant showed a log2FC above the cut-off values (Fig. 7*A* and Dataset S1). A comparison across genotypes of the log2FC values from the DEGs identified in the CO7-treated control plants further highlights the lack of response in the *lyka* and *lyr* mutants (Fig. 7*B*). Altogether, these results indicate that the *lyka* and *lyr* mutants of *M. paleacea* are equally unable to induce gene expression in response to CO7.

## Discussion

While single mutants in various *LYKs* in angiosperms have been observed to exhibit delayed or reduced colonization rates (10, 11, 35–38), none is completely defective for AM. In contrast, this work describes a single *LYK* mutant that displays an AM phenotype as strong as those observed in mutants of the CSSP in angiosperms (4, 5, 39–44) or *M. paleacea* (8, 19). The absence of AM in *lyka* is consistent with the AM-defective phenotypes described in the *M. truncatula lyk8/lyk9* (15) and in the *L. japonicus lys6/lys7* double mutants (14). This result obtained in a bryophyte species, combined with the phenotypes of the double mutants in legumes, corroborate the ancestral role of the LYK-I subclade in AM and substantiate the hypothesis that perception of fungal-derived molecule(s) by LysM-RLK is a prerequisite for AM establishment. Conversely, the loss of function of either *MpaLYKb* or *MpaLYKc* did not affect the establishment of AM. While no symbiotic function has yet been described for members of the LYK-III subclade (*MpaLYKc*), the co-orthologs of *MpaLYKb* (LYK-II subclade) in *L. japonicus*, *LjEPR3* and *LjEPR3a*, have been shown to play regulatory functions during the accommodation of AMF and rhizobia (45, 46). *LYK-II* genes are also upregulated during AM in a range of plant species, including monocots (47), dicots, and bryophytes (for review, see ref. 10). Interestingly, they are conserved in plants that form endosymbioses and are otherwise lost, a phylogenetic pattern that has been observed for genes of the CSSP (25). These two lines of evidence support the existence of a conserved function for members of the LYK-II subclade in endosymbioses. The persistence of AM formation in the *LYK-II* mutants of *M. paleacea, L. japonicus,* and rice suggests however that this function might come in addition to other signaling cues.

In addition to providing evidence supporting the involvement of MpaLYKa in AM, we demonstrate through measurement of cytosolic calcium influx that MpaLYKa is essential for both COs and LCOs perception. This result is in line with the MpLYK1-dependent ROS production in response to CO7 in *M. polymorpha*. This calcium influx following COs and LCOs perception in *M. paleacea* is independent of *SYMRK*, suggesting that it is distinct from the calcium spiking response observed in and around the nucleus, which strictly requires activation of the CSSP (48, 49). This observation is consistent with research in legumes, demonstrating the existence of an early calcium influx independent of the CSSP in response to Nod-LCOs (50) or CO8 and CO4 (51).

Perception of COs/LCOs in plants often involves heterocomplexes between LysM-RLKs of the LYK-I and LYR subclades (10) and complex formation between MpaLYKa and MpaLYR receptors has been reported (52). In angiosperms, the LYR subgroup has expanded, giving rise, for example, to four and eleven paralogs in *Brachypodium distachyon* and *M. truncatula*, respectively (10). The potential function of LYRs during AM has been extensively studied and single mutants have been tested for their symbiotic abilities in *M. truncatula* (*nfp* and *lyr4*) (13, 53), *L. japonicus* (*Ljlys11*) (54), *Parasponia andersonii* (*Pannfp1*, *Pannfp2*) (38), tomato (*sllyk10*) (11), Petunia (*Phlyk10*) (11), barley (*rlk2* and *rlk10*) (40), *B. distachyon* (*bdlyr1* and *bdlyr2*) (12) and rice (*Osnfr5*) (55). In addition, the double mutants *M. truncatula nfp/lyr4* (13) and *nfp/lyr1*, *L. japonicus lys11/nfr5* (54), *B. distachyon bdlyr1/bdlyr2* (12), and barley *rlk2/rlk10* (40) were tested. Most of the single mutants displayed wild-type level of colonization by AM fungi, with the exception of tomato and petunia single mutants, and even double mutants were either not or only partially affected in their AM fungi colonization levels. One hypothesis to explain these mixed results is the occurrence of significant variation in the number of LysM-RLKs between these species and possible genetic redundancy or variable contributions from the different paralogs in a species-specific manner. In *M. paleacea*, only one member of the *LYR* subgroup is found. Despite some notable differences, the phenotypes of *lyr* loss-of-function mutants in both this study and Tan et al. indicate that this receptor is not essential for AM establishment in *M. paleacea*. Our study found no difference between wild-type and *lyr* (Fig. 3 and *SI Appendix*, Figs. S3 and S7 and Table S1), while Tan et al. reported a strong impact of their *lyr* mutations on AM at low inoculum and a weak impact at higher inoculum (52). Differences in AMF strains, variable growth conditions, and/or mutation types might contribute to this discrepancy. In particular, the *lyr* mutations in the study of Tan et al. were generated using protospacers positioned after the transmembrane coding region (52). This could result in the production of a truncated MpaLYR lacking its kinase region, and such truncated receptor variants have been shown to exhibit dominant negative effects (56, 57). For example, expression of ERECTAΔKinase phenocopies the *erecta* loss-of-function mutant when expressed in wild-type *Arabidopsis* and exaggerates the growth defects of the null *erecta* plants (56).

Interestingly, MpaLYR is able to bind both short and long chain COs with high affinity. The competitive ability of Fuc/MeFuc-LCOs against CO5 for MpaLYR binding suggests that MpaLYR also functions as a receptor for LCOs. The biochemical properties of MpaLYR are thus reminiscent of those described for angiosperm LYR-IB receptors (10). This supports an evolutionary scenario in which receptors with specificity for LCOs, such as members of the LYR-IA (11, 58, 59) and LYR-IIIA (60, 61) subclades, and receptors with specificity for long-chain versus short-chain COs, such as members of the LYR-IIIC subclade (15, 62), have emerged after the LYR expansion in angiosperms. This could have led to a fine-tuning of the perception of symbionts and pathogens.

In contrast to MpaLYR, no direct binding activity toward COs could be detected in any of the MpaLYK receptors and a similar observation has been reported in *M. truncatula* concerning the ligand-binding abilities of LYR and LYK proteins (15). Whether MpaLYKa alone can serve as a receptor for yet unidentified LCOs structures remains unknown. However, MpaLYR and MpaLYKa are both required in *M. paleacea* for responses to CO7, CO4, and Fuc/MeFuc-LCOs (this study and 52). The absence of calcium spiking in response to *R. irregularis* germinated spore exudates (GSEs) in *lyr* single mutants reported by Tan et al. further suggests that MpaLYKa alone is not sufficient for the perception of molecules present in GSEs, including COs and LCOs (52). Given that MpaLYKa/MpaLYR are able to interact (52), this supports the existence of an MpaLYR/MpaLYK heterocomplex for CO/LCO perception with a binding property conferred by MpaLYR. Additionally, CO7-dependent gene reprogramming was entirely abolished in *lyr*, as it was in *lyka*. These results mirror the critical roles of both MpoLYK1 and MpoLYR in CO7-triggered ROS production and defense gene expression in the non-AM-host species *M. polymorpha* (22).

By functional analogy with bacterial Nod-LCOs that are essential for the nitrogen-fixing symbiosis in legumes, LCOs, and short-chain COs produced by AM fungi have been proposed to represent a blend of symbiotic signals that initiate symbiotic responses (52, 53, 63, 64). However, it remains unclear whether the perception of these molecules by the host is a prerequisite for AM establishment. Interestingly, other compounds, such as CO8, can induce similar molecular and cellular responses (15). Furthermore, large-scale analyses of exudates from sixty fungal species revealed that neither short-chain COs nor LCOs are specific to AM fungi (29, 65), raising questions about how plants can discriminate between beneficial and detrimental organisms based on this class of compounds alone. Our work in *M. paleacea* suggests that AM establishment can be uncoupled from COs and LCOs perception events and that MpaLYKa-dependent perception of other types of symbiont-derived signal(s) occurs. The suite of *M. paleacea* mutants developed here offer the opportunity to identify these signal(s) in the future.

The study of mutants in bryophyte species which have lost the ability to form symbiosis allowed identifying the ancestral function of LysM-RLK in plant immunity over the last decade (22, 52, 66). Our work extends this knowledge and proposes that LysM-RLK have been maintained in land plant genomes for almost half a billion years not only to perceive pathogens but as readers of the biotic environment, both for immunity and symbiosis.

## Materials and Methods

### Phylogeny.

To reconstruct the phylogeny of LysM-RLKs, a set of 102 protein sequences composed of 8 Marchantia genes and the set described by Buendia et al. supplemented with the *L. japonicus* EPR3 and EPR3a sequences (46) was aligned using MUSCLE (v3.8). The phylogeny was reconstructed using IQ-TREE v1.6.12 http://iqtree.cibiv.univie.ac.at/ with the VT+F+I+G4 model and support was provided with 1,000 ultrafast bootstrap replicates (67–69). The unrooted tree visualization was generated using iTOL (70) and adapted for presentation using InkScape.

### Plant Material, Growth Conditions, and Transformation.

For transformation, *M. paleacea* ssp*. paleacea* wild-type plants (18) were grown axenically in vitro from sterile gemmae on ½ strength Gamborg B5 (G5768, Sigma) medium supplemented with 1.4% agar (1330, Euromedex) for 3 to 4 wk in 16/8 h photoperiod at 22 °C/20 °C (61 µmol m^−2^ s^−1^; neon light, 60/36 W white Cooldaylight and pink Fluora, OSRAM). Approximately 20 plantlets were blended 15 s in a sterile 250 mL stainless steel bowl (Waring, USA) in 10 mL of OM51C medium [KNO_3_ (2 g L^−1^), NH_4_NO_3_ (0.4 g L^−1^), MgSO_4_·7H_2_O (0.37 g L^−1^), CaCl_2_·2H_2_O (0.3 g L^−1^), KH_2_PO_4_ (0.275 g L^−1^), L-glutamine (0.3 g L^−1^), casamino-acids (1 g L^−1^), Na_2_MoO_4_·2H_2_O (0.25 mg L^−1^), CuSO_4_·5H_2_O (0.025 mg L^−1^), CoCl_2_·6H_2_O (0.025 mg L^−1^), ZnSO_4_·7H_2_O (2 mg L^−1^), MnSO_4_·H_2_O (10 mg L^−1^), H_3_BO_3_ (3 mg L^−1^), KI (0.75 mg L^−1^), EDTA ferric sodium (36.7 mg L^−1^), myo-inositol (100 mg L^−1^), nicotinic acid (1 mg L^−1^), pyridoxine HCL (1 mg L^−1^), thiamine HCL (10 mg L^−1^)]. The blended plant tissues were transferred to 250 mL Erlenmeyers containing 50 mL of OM51C and kept at 22 °C/20 °C in the same room. 200 RPM for 3 d. Cocultures with *A. tumefaciens* (GV3101) containing constructs were initiated by adding 1 mL of saturated bacterial liquid culture (OD_600_ = 0.5 in 50 mL final), acetosyringone (100 µM final), and glutamine (0.5 g L^−1^ final). After 3 d, plant fragments were washed by decantation 3 times with water and plated on half-strength Gamborg containing 200 mg L^−1^ amoxicillin (Levmentin, Laboratoires Delbert, Fr.) and 10 mg L^−1^ Hygromycin B (3250, Euromedex) or 50 nM Chlorsulfuron (34322-100MG Sigma). Plant material is available in *SI Appendix*, Table S5.

For AM experiments, *M. paleacea* ssp*. paleacea* control and mutants were grown on a zeolite substrate (50% fraction 1.0 to 2.5 mm, 50% fraction 0.5 to 1.0 mm, Symbiom, Lanškroun, Czech Republic) in 7 × 7 × 8 cm pots with a density of five thalli per pot. Pots were watered once a week with Long Ashton medium containing 7.5 μM of phosphate (71) and grown with a 16/8 h photoperiod at 22 °C/20 °C (65 µmol m^−2^ s^−1^; neon light, 60/36 W white Cooldaylight, OSRAM). for at least 2 wk prior inoculation with AM fungi. For calcium measurement, similar conditions were applied with the exception of a higher plant density per pot and a growth duration of 4 wk or plants were grown axenically in vitro on 0.4% phytagel diluted in Long Ashton medium containing 7.5 μM of phosphate. For RNA Sequencing (RNAseq) experiments, plants were grown axenically in vitro on 0.4% phytagel diluted in Long Ashton medium containing 7.5 μM of phosphate for a duration of 4 wk.

### Cloning.

The Golden Gate modular cloning system was used to prepare the plasmids as described by Schiessl et al. (72). Level 1 and Level 2 empty vectors used in this study are listed in *SI Appendix*, Table S3 and held for distribution in the ENSA project core collection https://www.ensa.ac.uk/. Other Level 0, Level 1, and Level 2 vectors containing constructs are listed in *SI Appendix*, Table S3. Coding sequences were either domesticated in-house by PCR in the pUPD2 vector or synthesized and cloned into pMS (GeneArt, Thermo Fisher Scientific, Waltham).

### Transient Expression Assays in *N. benthamiana*.

*Agrobacterium tumefaciens* (GV3101) cultures carrying vectors of interest were grown under agitation overnight at 28 °C in liquid LB medium supplemented with adequate antibiotics. Cultures were washed twice by centrifugation at 8,000 g and resuspended in water supplemented with 50 µM acetosyringone. Bacteria concentrations were adjusted to OD_600_ = 0.2 per construct prior infiltration of the abaxial side of 2 mo old *N. benthamiana* leaves using a 1 mL needle-less syringe.

### Microscopy.

For microscopy analyses, mCherry and GFP signals were sequentially acquired 48 h after *N. benthamiana* transfection using a Leica SP8 confocal microscope mounted with a FLUOTAR VISIR 25×/0.95NA water immersion objective (Leica). GFP was excited at 488 nm and detected from 500 to 535 nm; monomeric Cherry was excited at 552 nm and detected from 560 to 610 nm. Images were processed using LasX (Leica).

### Binding Assays.

Preparation of microsomal fraction was performed as previously described by Girardin et al. (11). CO5-biotin (CO5*) and CO7-biotin (CO7*) were synthesized as detailed by Cullimore et al. (59) and Ding et al. (12) respectively. Binding assays were conducted using CO5* or CO7* and competitors, mixed with the appropriate microsomal fraction in binding buffer (25 mM NaCacodylate pH 6, 1 mM MgCl_2_, 1 mM CaCl_2_, 250 mM sucrose, and protease inhibitors). Samples were incubated for 30 min on ice and subsequently centrifuged for 30 min at 31,000 g and 4 °C. Pellets were resuspended in IP buffer (25 mM Tris-HCl pH7.5, 150 mM NaCl, 10% glycerol, supplemented with protease inhibitors). Proteins were solubilized in IP buffer supplemented with 0.2% DDM for 1 h at 4 °C and centrifuged during 30 min at 31,000 g and 4 °C. The supernatant was retrieved, and mCherry-tagged receptors were immunoprecipitated using RFP-trap magnetic agarose beads (ChromoTek). Beads were washed twice with IP buffer, and proteins were extracted in Laemmli buffer. Proteins were separated by SDS-PAGE and transferred onto a nitrocellulose membrane using the Trans-Blot system (Bio-rad) according to the manufacturer’s instructions. Membranes were blocked for 1 h with 5% fat-free milk in Tris-buffer Saline (TBS) supplemented with 0.1% Tween-20 (TBS-T). Membranes were incubated for 1 h with the appropriate antibodies: rabbit αRFP (73) (1:5,000), αRabbit-Hrp (Millipore, 1:20,000), and streptavidin-Hrp (Invitrogen, 1:3,000). Hrp activity was detected using Clarity Western ECL substrate (Bio-Rad) and observed with a ChemiDoc imager (Bio-Rad).

### Generation of CRISPR Mutants in *M. paleacea* ssp. *paleacea*.

Constructs containing the *A. thaliana* codon-optimized SpCas9 (74) under the control of *MpoEF1α* promoter (32) and single guide RNAs (*SI Appendix*, Tables S1 and S2) under the *M. polymorpha* or *M. paleacea* U6 promoters were introduced in wild-type *M. paleacea.* Independent transformants for *lyka, lykb, lykc,* and *lyr* were selected for AM phenotyping. A *M. paleacea* line expressing the calcium reporter aequorin (AEQ) under the control of *MpoEF1α* promoter was retransformed with the CRISPR/Cas9 vectors, modified to contain a secondary selection marker (ChlorsulfuronR). Two independent mutant lines per receptor were used for calcium influx assays.

### Mycorrhiza Tests.

Each pot containing 5 plants was inoculated with ~1,000 sterile spores of *Rhizophagus irregularis* DAOM 197198 (Agronutrition, France) and grown with a 16/8 h photoperiod (65 µmol m^−2^ s^−1^; neon light, 60/36 W white Cooldaylight, OSRAM) at 22 °C/20 °C. Plant trays were covered with a transparent lid and maintained under high humidity for the duration of the experiment. For the nursing experiment, thalli of empty-vector control and *lyka* were transplanted in proximity to wild-type plants inoculated with *R. irregularis* spores more than 10 wk before. Six- or ten-weeks postinoculation (for nursing), thalli were cleared of chlorophyll using ethanol 100% for 1 d before storage in an EDTA (0.5 mM). The cleared thalli were scanned using an EPSON 11000XL. The exclusion zone was then determined by measuring the distance between apical notches and the AMF-colonized zone, which is colored with a purple/brown pigment (2). Higher exclusion zone values than the control denote lower AM colonization than the control. Large-scale mycorrhiza assays were run independently twice.

For imaging, cleared thalli of control and mutants were embedded in 6% agarose and 100 µm thick transversal and longitudinal sections were prepared using a Leica VT1000s vibratome. Sections were incubated overnight in 10% KOH (w/v) prior to three washes in PBS. Fungal membranes were stained overnight using 1 µg mL^−1^ WGA-Alexa Fluor 488 (Sigma) diluted in PBS. Pictures were taken with a Zeiss AxioZoom V16 binocular using a PlanApo Z 0.5× objective and the ZEN software suite with similar settings. Alexa Fluor was excited between 450 and 490 nm and emission collected between 500 and 550 nm.

### Calcium Influx Measurements.

Three to four weeks old plants were submerged in 2.5 μM coelenterazine-h (Interchim) diluted in water for a minimum of 16 h at room temperature and in the dark. Samples were then transferred in a Berthold Sirius luminometer before treatments with a 250 µL aqueous solution of 1 µM CO7 dilutions, 1 µM CO4 (Elicityl, Crolles, France) or a 1 µM mixture of fucosyl/methylfucosyl LCO-V (C18:1, Fuc/MeFuc), 1 µM nonsulfated LCO-IV (C18:1) and 1 µM sulfated LCO-IV (C18:1, S) (29, 75). Luminescence was continuously recorded for 15 min using a 1 s interval. An equal volume of 2× lysis buffer [20% EtOH, 10 mM CaCl_2_, 2% NP-40] was injected to discharge the total luminescence left and light was collected for an additional 5 min. Calcium concentrations were calibrated as previously done (76). Graphs were prepared using RStudio and the ggplot2 package by plotting signals from 10 to 900 s after treatment, prior to assembly using the InkScape software. Areas under the curve from 10 to 900 s were calculated minus the baseline value, defined as the lowest value present in each trace. A minimum of three traces for each experiment were recorded.

### RNA Extraction and RNAseq.

Gemmae from control Cas9, *lyka-1,* and *lyr-1* were surface sterilized with a nylon cell strainer 40 µm (Falcon) in a bleach solution (~0.27%) for 1 min and washed in water twice before plating on ½ strength Gamborg B5 media and grown with a 16/8 h photoperiod at 22 °C/20 °C. Plants were treated or not with 1 µM elicitors for 1 h before being frozen in liquid nitrogen. Tissues were ground with mortar and pestle using liquid nitrogen prior total RNAs extraction using the RNA Plant and Fungi NucleoSpin™ (Macherey-Nagel™). DNA was eliminated using the Macherey-Nagel™ Kit rDNase. RNAs quality and quantity were determined by Nanodrop. Total RNAs were sent for sequencing to Genewiz/Azenta (Leipzig, Germany). Illumina libraries were prepared with the NEBnext ultra II RNA directional kit and sequenced on a NovaSeq platform. The samples were sequenced using a 2 × 150 Pair-End reads configuration.

### RNAseq Analysis.

RNASeq raw reads from all described conditions were mapped against the genome of *M. paleacea* (18) and counted using the Nextflow v23.10.0 (77) pipeline NF-CORE/RNASeq v3.14 (78) with the options star_salmon to align and quantify reads, as well as “-nextseq 30 -length 50” as extra parameters of TrimGalore v0.6.7 (79) to remove reads with quality lower than 30 or a length lower than 50 bp. Ribosomal RNA were also removed through the option “-remove_ribo” using SortMeRNA v4.3.4 (80). The pipeline was run under the GenoToul configuration available here: https://github.com/nf-core/configs/blob/master/docs/genotoul.md and used the following software and languages: bedtools v2.30.0 (81), R v4.0.3, v4.1.1, and v4.2.1 (82), DESEQ2 v1.28.0 (83); dupradar v1.28.0 (84), fastqc v 0.12.1 (85), fq v0.9.1 https://github.com/stjude-rust-labs/fq, gffread v0.12.1 (86), perl v5.26.2 (87), python v3.9.5 (88), rsem v1.3.1 (89), STAR v2.7.10a (90), picard v3.0.0 (91), qualimap v2.3 (92), rseqc v5.02 (93), salmon v1.10.1 (94), summarizedExperiment v1.24.0 (95), samtools v1.16.1 (96), stringtie v2.2.1 (97), tximeta v1.12.0 (98), UCSC v377, and v445 https://github.com/ucscGenomeBrowser/kent. The R package DESeq2 v1.42.1 was used with default parameters for the differential gene expression analysis. Venn diagram was constructed using the https://jvenn.toulouse.inrae.fr/ webserver with size modification in Inkscape. Enriched IPR terms were determined using the R package clusterProfiler v4.12.3, the DEGs with a log2FC threshold of |1| as foreground and genes subjected DEG analysis (i.e., genes that were retained after removing the low counts) as background. A template script is available at https://gist.github.com/LRSV-MMbengue/beb55a608d6053c9aaa9257993c6bf6b.

### Statistical Analyses.

To evaluate differences versus control, Student’s *t* test or ANOVA followed by Tukey’s HSD as the post hoc test was performed using R.

## Supplementary Material

Appendix 01 (PDF)

Dataset S01 (XLSX)

Dataset S02 (XLSX)

## Data Availability

Raw sequencing data for the RNASeq analysis have been deposited at the European Nucleotide Archive under the code PRJEB89852 and accessible at https://www.ebi.ac.uk/ena/browser/view/PRJEB89852 (99). All other data are included in the article and/or supporting information.
