# Supplementary material for: A plant Lysin Motif Receptor-Like Kinase plays an ancestral function in mycorrhiza
Source: Proc Natl Acad Sci U S A. 2025 Jun 11;122(24):e2426063122. doi: 10.1073/pnas.2426063122 (PMC12184373; doi:10.1073/pnas.2426063122)
Supplement: Supplementary file 1 — Appendix 01 (PDF) [file pnas.2426063122.sapp.pdf]

PNAS

Supporting Information for
A plant Lysin Motif Receptor-Like Kinase plays an ancestral function in mycorrhiza

Eve Teyssier[1], Sabine Grat[1], David Landry[2], Mathilde Ouradou[1], Mélanie K. Rich[1], Sébastien Fort[3], Jean Keller[1], Benoit Lefebvre[2], Pierre-Marc Delaux[1*], Malick Mbengue[1*]

Malick Mbengue
Email: malick.mbengue@univ-tlse3.fr

Pierre-Marc Delaux:
Email: pierre-marc.delaux@cnrs.fr

This PDF file includes:

Figures S1 to S10
Tables S1 to S3
Datasets S1 to S2

Supporting information

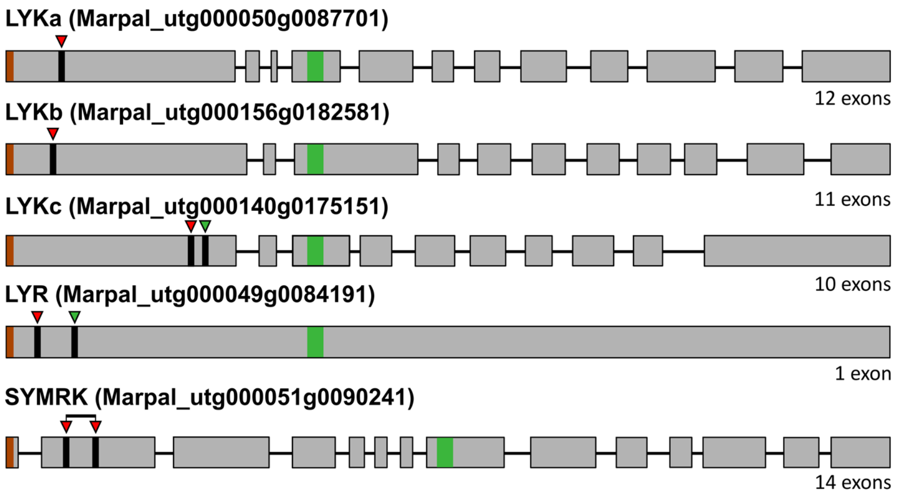

Fig S1. Protospacers positions for generating loss-of-function RLK mutants in *M. paleacea*. Gene structure representation of the four *M. paleacea LysM-RLKs* and *SYMRK:* exons (grey boxes), introns (black line), signal peptides and transmembrane domain coding sequences are highlighted in brown and green, respectively. Arrowheads point at protospacers positions used for generating CRISPR/Cas9 loss-of-function mutants. For *SYMRK*, a polycistronic RNA construct was designed to generate two guide RNAs.

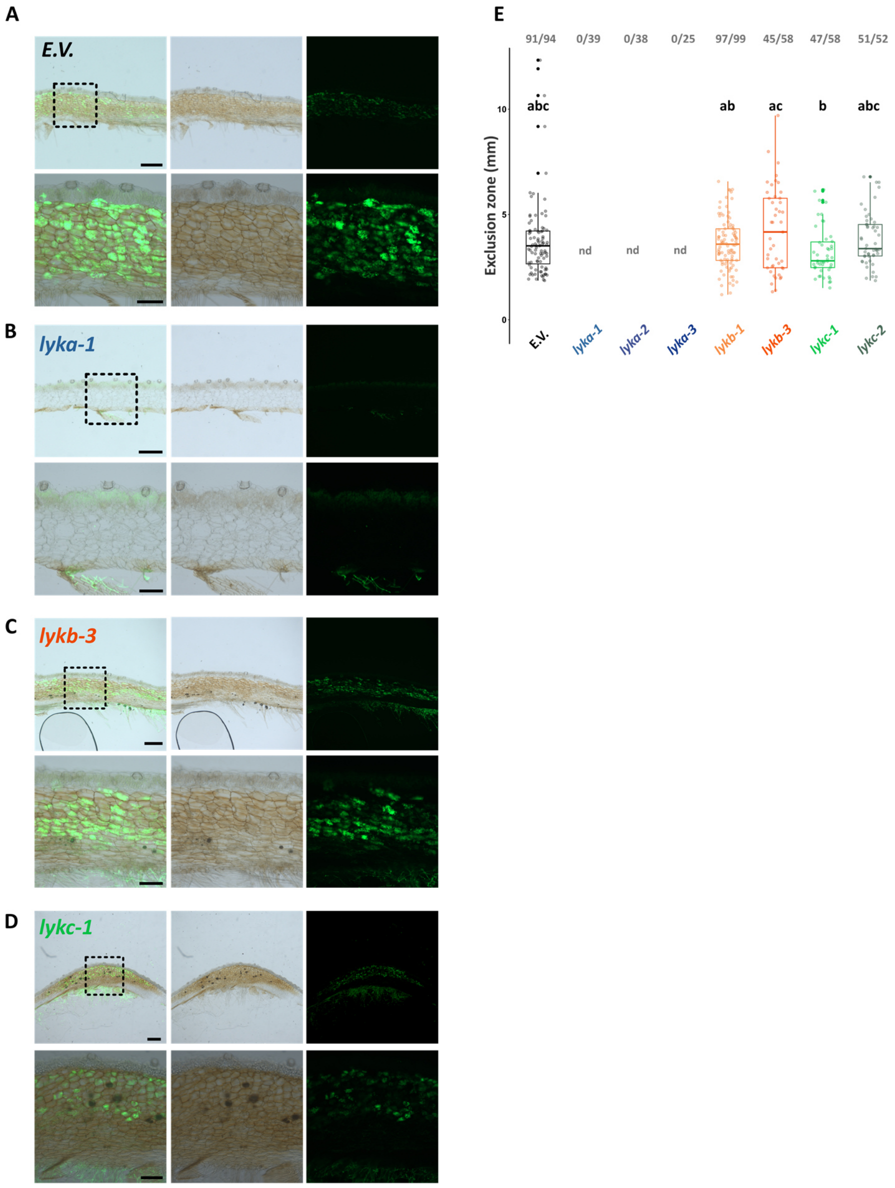

Fig S2. Longitudinal sections of *M. paleacea lyk* mutants and exclusion zone measurements. (**A-D**) Representative images of longitudinal sections of *M. paleacea* control (E.V.) or *lyk* mutants, as indicated, six weeks after inoculation with *R. irregularis*. Left panels are overlays of bright field (middle panels) and fluorescent (right panels) images. WGA coupled to Alexa Fluor 488 was used to detect fungal structures. Dashed line delimited insets are enlarged underneath the original images. Scale bars are 500 µm and 200 µm for insets. (**E**) Quantitative analysis of the exclusion zone length on mycorrhizal plants for control (E.V.) and loss-of-function *lyk* mutants. For each genotype, fractions in bold grey represent mycorrhizal thalli over total thalli assessed. Different letters indicate differences to control inferred by ANOVA followed by Tukey's HSD post-hoc test (p-value < 0.05). Control (E.V.) values are shared with **Fig S3** and statistical analysis was performed on the whole dataset. "nd" stands for not determined.

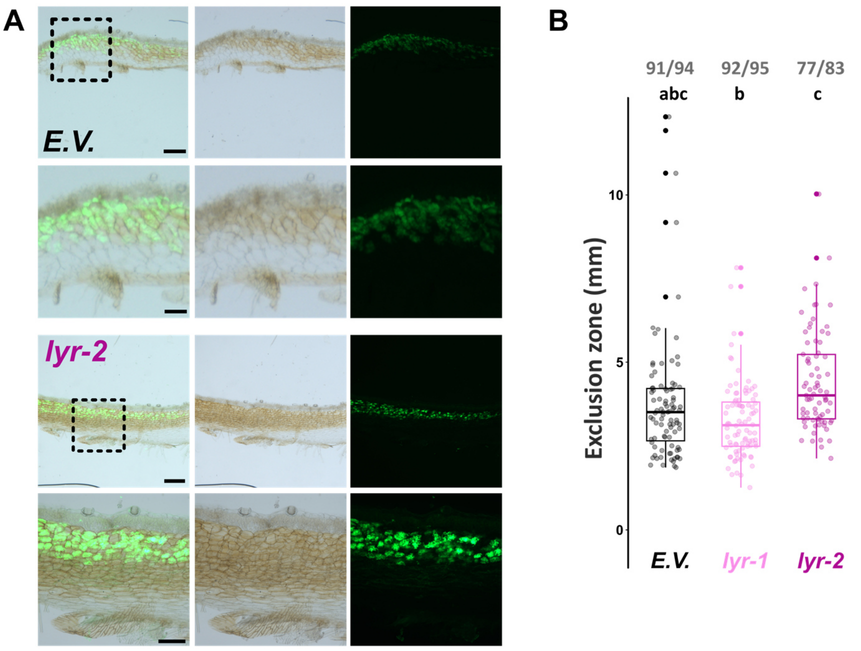

Fig S3. Longitudinal sections of *M. paleacea lyr* mutant and exclusions zones measurements. (**A**) Representative images of longitudinal sections of *M. paleacea* control (E.V.) or the *lyr-2* mutants six weeks after inoculation with *R. irregularis*. Left panels are overlays of bright field (middle panels) and fluorescent (right panels) images. WGA coupled to Alexa Fluor 488 was used to detect fungal structures. Dashed line delimited insets are enlarged underneath the original images. Scale bars are 500 µm and 200 µm for insets. (**B**) Quantitative analysis of the exclusion zone length on mycorrhizal plants for control (E.V.) and two independent *lyr* mutants. For each genotype, fractions in bold grey represent mycorrhizal thalli over total thalli assessed. Different letters indicate differences to control inferred by ANOVA followed by Tukey's HSD post-hoc test (p-value < 0.05). Control (E.V.) values are shared with **Fig S2** and statistical analysis was performed on the whole dataset.

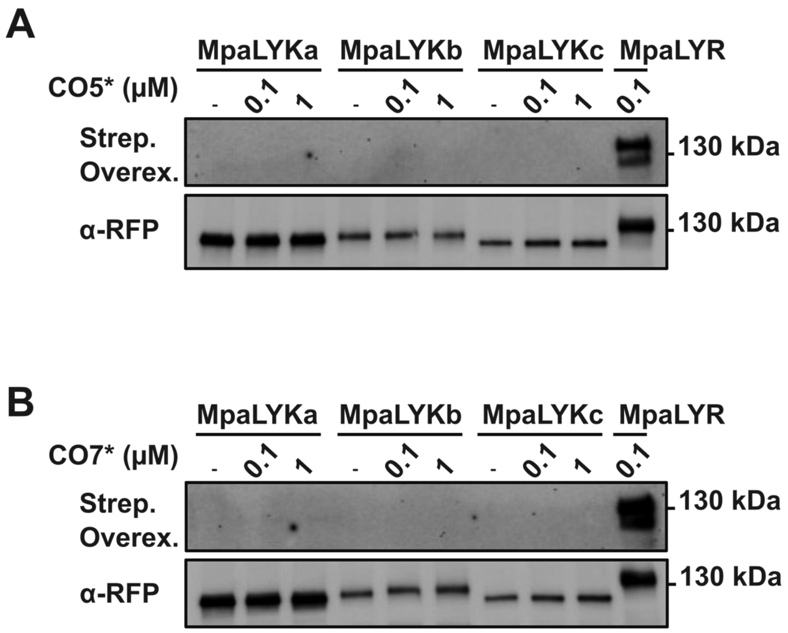

Fig S4. MpaLYR has high affinity for chito- and lipochito-oligosaccharides in *M. paleacea.* (**A**) and (**B**) are identical to the panels shown in **Fig 4B** and **Fig 4D**, respectively, but with over-exposition of the streptavidin-HRP detection. RFP detection was not modified.

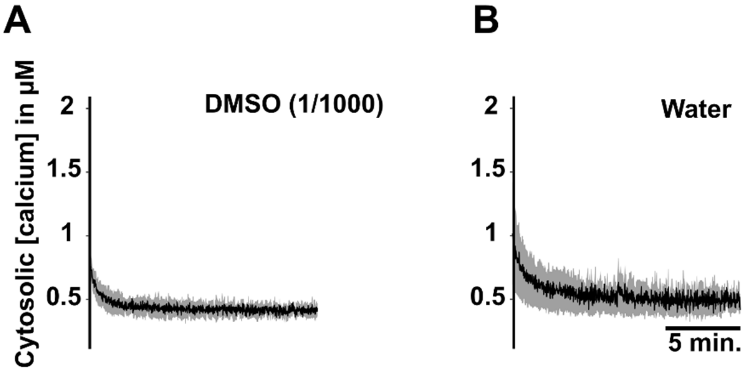

Fig S5. DMSO and water alone do not elicit calcium influx in *M. paleacea.* Cytosolic calcium concentration variations in *M. paleacea* control plants (AEQ-cas9) in response to (**A**) 0.1% DMSO diluted in water or (**B**) water alone, over a time course of 15 min. (**A-B**) Each trace represents the mean (line) ± standard deviation (shading) from at least three replicates.

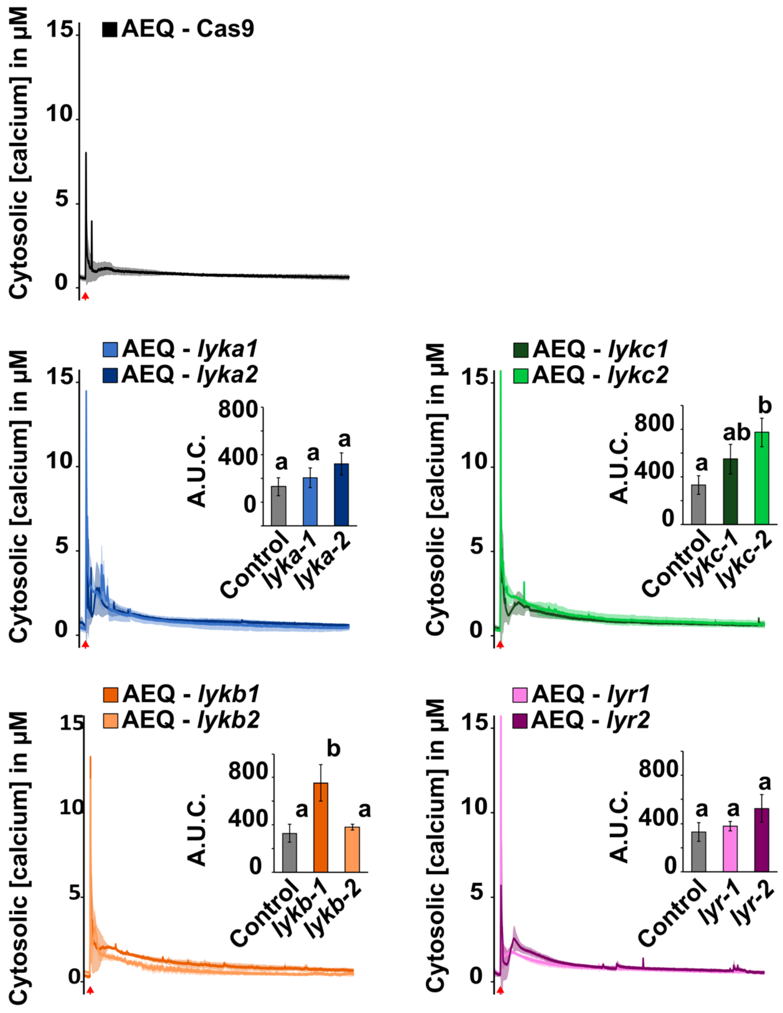

Fig S6. ***M. paleacea*** **LysM-RLK mutants produce a calcium influx in response to hydrogen peroxide.** Cytosolic calcium concentration variations in *M. paleacea* control (AEQ-cas9) or two independent mutant lines for each *LysM-RLK*, as indicated, and treated with 1mM hydrogen peroxide diluted in water. Each trace represents the mean (line) ± standard deviation (shading) from three replicates over a time course of 15 min. Bar graph insets represent the area under the curves (A.U.C.) corrected for the baseline and different letters indicate significant differences versus control inferred by ANOVA and Tukey's HSD post-hoc test (p-value < 0.05).

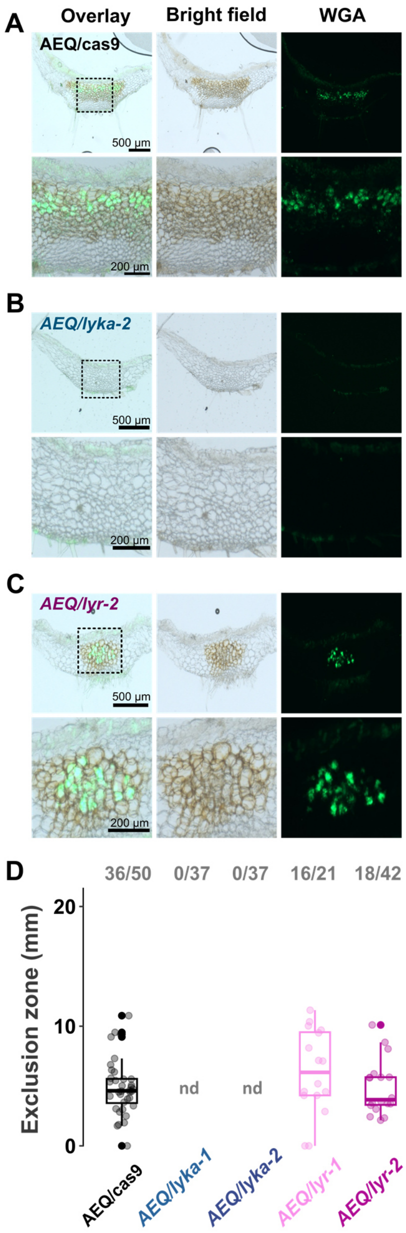

Fig S7. *lyka* and *lyr* mycorrhizal phenotypes in the *M. paleacea* aequorin-expressing background. (**A-C**) Representative images of transversal sections of *M. paleacea* control (AEQ/cas9), one AEQ/*lyka* and one AEQ/*lyr* mutant, six weeks after inoculation with *R. irregularis*. Left panels are overlays of bright field (middle panels) and fluorescent (right panels) images. Wheat germ agglutinin (WGA) coupled to Alexa Fluor 488 was used to detect fungal structures. Dashed line delimited insets are enlarged underneath the original images. Scale bars are 500 µm and 200 µm for insets. (**D**) Quantitative analysis of the exclusion zone length on mycorrhizal plants for control (AEQ/cas9) and two independent *lyka* and *lyr* mutants. For each genotype, fractions in bold grey represent mycorrhizal thalli over total thalli assessed.

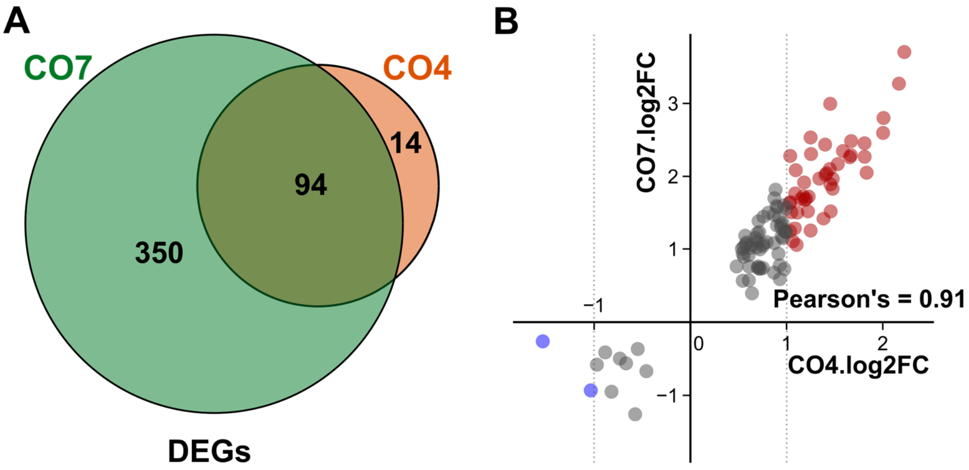

Fig S8. Transcriptomics response to CO4 is a subset of the CO7 response. (**A**) Venn diagram of DEGs in control plants after one-hour CO7 or CO4 treatment versus water (p-value adjusted < 0.05). (**B**) Scatter plot of the log2 fold changes from the 108 DEGs in response to CO4 (x-axis) against their corresponding log2 fold changes in response to CO7 (y-axis). The 45 up-regulated and 2 down-regulated genes in response to CO4 (|log2FC| ≥ 1 – dashed grey bars) are highlighted in red and blue, respectively. The Pearson's correlation coefficient between CO7 and CO4 log2FC values is indicated.

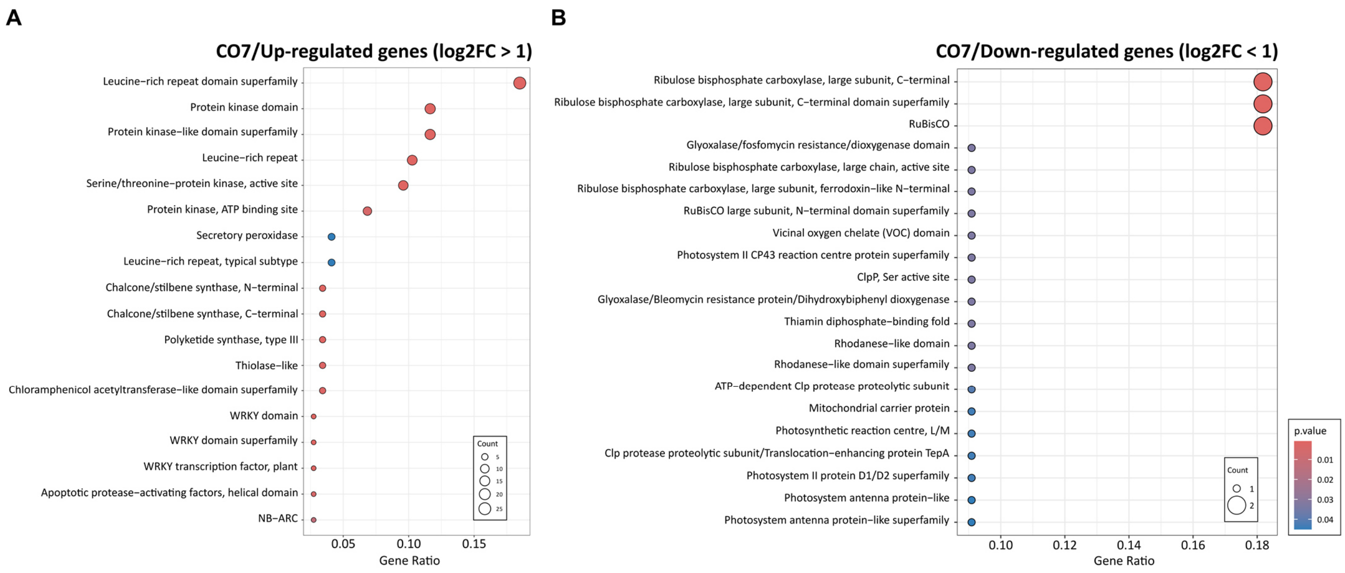

Fig S9. InterPro terms enrichment analyses in response to CO7. InterPro terms enrichment among up-regulated (**A**) and down-regulated (**B**) genes after 1µM CO7 treatment. DEGs are statistically significant (p-value adjusted < 0.05 and |log2FC| ≥ 1).

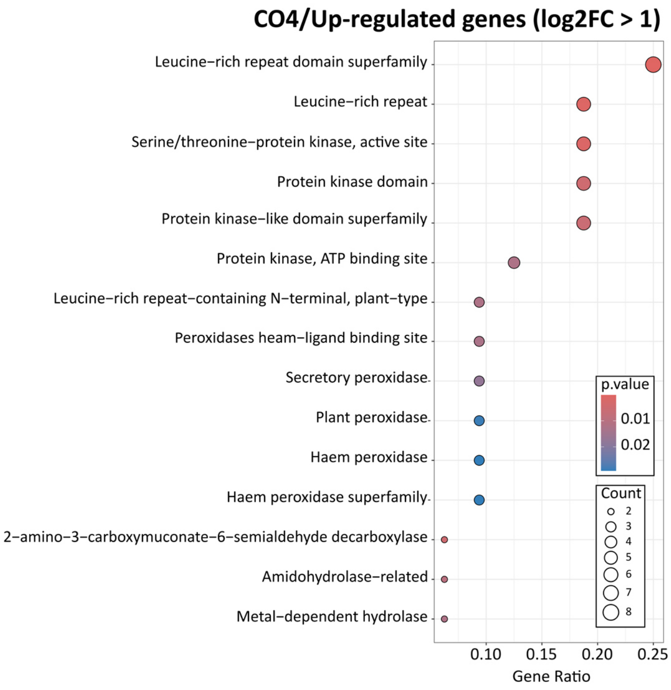

Fig S10. InterPro terms enrichment analysis in response to CO4. InterPro terms enrichment among up-regulated genes after 1μM CO4 treatment. DEGs are statistically significant (p-value adjusted < 0.05 and |log2FC| ≥ 1).

S1 Table. List of protospacers sequences used for gene editions.

	GENBANK	GENE	PROTOSPACER #1	PROTOSPACER #2	MUTANT	MUTATION
Protospacers sequences (w/o PAM)	MUAA02000050.1	Marpal_utg000050g0087701	TGATACTCTCCTAGCTCTGG	-	*lyka-1*	+A/frameshift at AA57 leading to STOP
					lyka-2	+A/frameshift at AA57 leading to STOP
					lyka-3	+A/frameshift at AA57 leading to STOP
	MUAA02000155.1	Marpal_utg000156g0182581	CGGAGCGGATTAATCAACTG	-	*lykb-1*	+T/frameshift A27 leading to STOP
					lykb-2	19bp deletion/frameshit AA27 leading to STOP
	MUAA02000139.1	Marpal_utg000140g0175151	GGACCGTTACGATGCGGATA	-	*lykc-1*	+T/frameshift at AA180 leading to STOP
			-	GCTCGGGAAGTAGCTGACAT	*lykc-2*	+G insertion/frameshift at AA192 leading to STOP
	MUAA02000049.1	Marpal_utg000049g0084191	ACTCGTGCTCAACAGGTCTA	-	*lyr-1*	+T/frameshift at AA26 leading to STOP
			-	TTACAAAGACATTCCCGCTG	*lyr-2*	+A/frameshift at AA61 leading to STOP
	MUAA02000050.1	Marpal_utg000050g0087701	TGATACTCTCCTAGCTCTGG	-	*AEQ-lyka-1*	+T/frameshit AA57 leading to STOP
					AEQ-lyka-2	+T/frameshit AA57 leading to STOP
	MUAA02000155.1	Marpal_utg000156g0182581	CGGAGCGGATTAATCAACTG	-	*AEQ-lykb-1*	+T/frameshift at AA27 leading to STOP
					AEQ-lykb-2	+T/frameshift at AA27 leading to STOP
	MUAA02000139.1	Marpal_utg000140g0175151	GGACCGTTACGATGCGGATA	-	*AEQ-lykc-1*	7pb insertion/frameshift at AA179 leading to STOP
					AEQ-lykc-2	+G/frameshift at AA179 leading to STOP
	MUAA02000049.1	Marpal_utg000049g0084191	ACTCGTGCTCAACAGGTCTA	-	*AEQ-lyr-1*	+T/frameshift at AA26 leading to STOP
					AEQ-lyr-2	+T/frameshift at AA26 leading to STOP
	MUAA02000051.1	Marpal_utg000051g0090241	CGGGATCAGATTTAAGCCTG	TAGCGTATAAAAAGGTCCCG	*AEQ-symrk-1*	80bp deletion/frameshift at AA81 leading to STOP
					AEQ-symrk-2	80bp deletion/frameshift at AA81 leading to STOP

S2 Table. List of primers for genotyping edited lines.

	GENBANK	GENE CODES	PRIMER #1	PRIMER #2	TARGET GENE
Primers for gRNA cloning	MUAA02000050.1	Marpal_utg000050g0087701_ g1	TCTCGTGATACTCTCCTAGCTCTGG	AAACCCAGAGCTAGGAGAGTATCAC	*LYKa*
	MUAA02000155.1	Marpal_utg000156g0182581_g1	TCTCGCGGAGCGGATTAATCAACTG	AAACCAGTTGATTAATCCGCTCCGC	*LYKb*
	MUAA02000139.1	Marpal_utg000140g0175151_g1	TCTCGGGACCGTTACGATGCGGATA	AAACTATCCGCATCGTAACGGTCCC	*LYKc*
	MUAA02000139.1	Marpal_utg000140g0175151_g2	TCTCGGCTCGGGAAGTAGCTGACAT	AAACATGTCAGCTACTTCCCGAGCC	*LYKc*
	MUAA02000049.1	Marpal_utg000049g0084191_g1	TCTCGACTCGTGCTCAACAGGTCTA	AAACTAGACCTGTTGAGCACGAGTC	*LYR*
	MUAA02000049.1	Marpal_utg000049g0084191_g2	TCTCGTTACAAAGACATTCCCGCTG	AAACCAGCGGGAATGTCTTTGTAAC	*LYR*
Genotyping primers	MUAA02000050.1	Marpal_utg000050g0087701	TGAGAATTCGCCTGTAGTTTG	ACCTTTCTGGGGGATAAAGATGA	*LYKa*
	MUAA02000155.1	Marpal_utg000156g0182581	ATGAGAGCGAGGAGTCCCGTA	GAAGTTGAGCGGGATGAAGAGGT	*LYKb*
	MUAA02000139.1	Marpal_utg000140g0175151	GTTTCAGACATCATGGAACAAAAAG	CCGTTCAACCGATCATAGTAC	*LYKc*
	MUAA02000049.1	Marpal_utg000049g0084191	AGGTGATTTCAAAGTGGGAGAC	TCCAGAGTGTTGTTGCTGAG	*LYR*
	MUAA02000051.1	Marpal_utg000051g0090241	AATGGAGGACACAGGAGAAGC	AACCCAAGACAAAACCAGTAAGG	*SYMRK*

S3 Table. List of constructs and selected lines used in this study.

CONSTRUCT	SOURCE	IDENTIFIER	DESCRIPTION
**BIOLOGICAL SAMPLES**			
Marchantia paleacea	Available here	N/A	wild-type
Marchantia paleacea Line 132.2	Rich et *al.* 2021	N/A	Transformed with an HygroR empty vector control
M. paleacea (lyka)	This paper	N/A	Mutant in wild-type background
M. paleacea (lykb)	This paper	N/A	Mutant in wild-type background
M. paleacea (lykc)	This paper	N/A	Mutant in wild-type background
M. paleacea (lyr)	This paper	N/A	Mutant in wild-type background
M. paleacea (AEQ-Cas9)	This paper	N/A	Mutant in aequorin-expressing background
M. paleacea (AEQ-lyka)	This paper	N/A	Mutant in aequorin-expressing background
M. paleacea (AEQ-lykb)	This paper	N/A	Mutant in aequorin-expressing background
M. paleacea (AEQ-lykc)	This paper	N/A	Mutant in aequorin-expressing background
M. paleacea (AEQ-lyr)	This paper	N/A	Mutant in aequorin-expressing background
M. paleacea (AEQ-symrk)	This paper	N/A	Mutant in aequorin-expressing background
**RECOMBINANT DNA**			
pUPD2 - AEQUORIN (B3-B5)	This paper	N/A	Golden gate Level 0 - CDS
pMS - MpalLYKa	This paper	N/A	Golden gate Level 0 - CDS
pMS - MpalLYKb	This paper	N/A	Golden gate Level 0 - CDS
pMS - MpalLYKc	This paper	N/A	Golden gate Level 0 - CDS
pMS - MpalLYR	This paper	N/A	Golden gate Level 0 - CDS
mCherry C-terminal tag	Addgene	pICSL50004	Golden gate Level 0 - CDS
pro35S - short	Addgene	pICH51277	Golden gate Level 0 - promoter
proMpolEF1a	Althoff et *al.* (2013)		Golden gate Level 0 - promoter
proAtUBQ10 promoter	This paper	N/A	Golden gate Level 0 - promoter
35S terminator	Addgene	pICH41414	Golden gate Level 0 - terminator
Nos terminator	Addgene	pICH41421	Golden gate Level 0 - terminator
pL1F2 - proAtUBI10::MpalLYKA-mcherry-35Ster	This paper	N/A	Golden gate Level 1 for expression in *N. benthamiana*
pL1F2 - proAtUBI10::MpaLYKB-mcherry-35Ster	This paper	N/A	Golden gate Level 1 for expression in *N. benthamiana*
pL1F2 - proAtUBI10::MpaLYKC-mcherry-35Ster	This paper	N/A	Golden gate Level 1 for expression in *N. benthamiana*
pL1F2 - proAtUBI10::MpaLYR-mcherry-35Ster	This paper	N/A	Golden gate Level 1 for expression in *N. benthamiana*
pL1R1 - pro35S::HygroR-Noster	ENSA	EC15030	Golden gate Level 1 - Selection marker
L1-R1 - p35S-ChlorosulfuronR_V2-35Ster	ENSA	1PMD1030	Golden gate Level 1 - Selection marker
pL1F2 - proMpolEF1a::AEQUORIN-Noster	This paper	N/A	Golden gate Level 1
pL1-R2 - pMpaEF1a:AtCas9:NLS-35Ster	ENSA	1PMD527	Golden gate Level 1
pL1-R3 - pMpoU6-1:sgRNA-tRNAP	Rich et *al.* 2021	1PMD97	Golden gate Level 1
pL2b - Hygro-MpalLYKa_guide1	This paper	N/A	Golden gate Level 2 for expression in *M. paleacea*
pL2b - Hygro-MpalLYKb_guide1	This paper	N/A	Golden gate Level 2 for expression in *M. paleacea*
pL2b - Hygro-MpalLYKc_guide1	This paper	N/A	Golden gate Level 2 for expression in *M. paleacea*
pL2b - Hygro-MpalLYKc_guide2	This paper	N/A	Golden gate Level 2 for expression in *M. paleacea*
pL2b - Hygro-MpalLYR_guide1	This paper	N/A	Golden gate Level 2 for expression in *M. paleacea*
pL2b - Hygro-MpalLYR_guide2	This paper	N/A	Golden gate Level 2 for expression in *M. paleacea*
pL2b - Chlorosulfuron-MpalLYKa_guide1	This paper	N/A	Golden gate Level 2 for expression in *M. paleacea*
pL2b - Chlorosulfuron-MpalLYKb_guide1	This paper	N/A	Golden gate Level 2 for expression in *M. paleacea*
pL2b - Chlorosulfuron-MpalLYKc_guide1	This paper	N/A	Golden gate Level 2 for expression in *M. paleacea*
pL2b - Chlorosulfuron-MpalLYR_guide1	This paper	N/A	Golden gate Level 2 for expression in *M. paleacea*
pL2b - Chlorosulfuron-MpalSYMRK_guide1&2	This paper	N/A	Golden gate Level 2 for expression in *M. paleacea*
pL2b - HygroR - proMpolEF1a::AEQUORIN-Noster	This paper	N/A	Golden gate Level 2 for expression in *M. paleacea*
pAtFLS2::AtFLS2–3xmyc-GFP-35Ster	Robatzek et al., 2006	N/A	

Dataset S1. List of differentially regulated genes in response to CO7 and CO4 in *M. paleacea* WT and CO7 for *lyka* and *lyr*. (Separate file).

Dataset S2. InterPro terms enrichment analyses in response to CO7 and CO4. (Separate file).
